# Double-Locking Mechanism of Self-Compatibility in *Arabidopsis thaliana*: The Synergistic Effect of Transcriptional Depression and Disruption of Coding Region in the Male Specificity Gene

**DOI:** 10.3389/fpls.2020.576140

**Published:** 2020-09-11

**Authors:** Keita Suwabe, Kaori Nagasaka, Endang Ayu Windari, Chihiro Hoshiai, Takuma Ota, Maho Takada, Ai Kitazumi, Hiromi Masuko-Suzuki, Yasuaki Kagaya, Kentaro Yano, Takashi Tsuchimatsu, Kentaro K. Shimizu, Seiji Takayama, Go Suzuki, Masao Watanabe

**Affiliations:** ^1^ Graduate School of Bioresources, Mie University, Tsu, Japan; ^2^ Department of Plant and Soil Science, Texas Tech University, TX, United States; ^3^ Graduate School of Life Sciences, Tohoku University, Sendai, Japan; ^4^ Life Science Research Center, Mie University, Tsu, Japan; ^5^ School of Agriculture, Meiji University, Kawasaki, Japan; ^6^ Graduate School of Science, The University of Tokyo, Tokyo, Japan; ^7^ Department of Evolutionary Biology and Environmental Studies, University of Zurich, Zurich, Switzerland; ^8^ Kihara Institute for Biological Studies, Yokohama City University, Yokohama, Japan; ^9^ Graduate School of Agricultural and Life Sciences, The University of Tokyo, Tokyo, Japan; ^10^ Division of Natural Science, Osaka Kyoiku University, Kashiwara, Japan

**Keywords:** *Arabidopsis thaliana*, artificial chimeric gene, evolutionary process, flower development, promoter activity, self-compatibility, *S*-locus protein 11/*S*-locus cysteine rich protein gene

## Abstract

Self-compatibility in *Arabidopsis thaliana* represents the relatively recent disruption of ancestral obligate cross pollination, recognized as one of the prevalent evolutionary pathways in flowering plants, as noted by Darwin. Our previous study found that inversion of the male specificity gene (*SP11*/*SCR*) disrupted self-incompatibility, which was restored by overexpressing the *SCR* with the reversed inversion. However, *SCR* in *A. thaliana* has other mutations aside from the pivotal inversion, in both promoter and coding regions, with probable effects on transcriptional regulation. To examine the functional consequences of these mutations, we conducted reciprocal introductions of native promoters and downstream sequences from orthologous loci of self-compatible *A. thaliana* and self-incompatible *A. halleri*. Use of this inter-species pair enabled us to expand the scope of the analysis to transcriptional regulation and deletion in the intron, in addition to inversion in the native genomic background. Initial analysis revealed that *A. thaliana* has a significantly lower basal expression level of *SCR* transcripts in the critical reproductive stage compared to that of *A. halleri*, suggesting that the promoter was attenuated in inducing transcription in *A. thaliana*. However, in reciprocal transgenic experiments, this *A. thaliana* promoter was able to restore partial function if coupled with the functional *A. halleri* coding sequence, despite extensive alterations due to the self-compatible mode of reproduction in *A. thaliana*. This represents a synergistic effect of the promoter and the inversion resulting in fixation of self-compatibility, primarily enforced by disruption of *SCR*. Our findings elucidate the functional and evolutionary context of the historical transition in *A. thaliana* thus contributing to the understanding of the molecular events leading to development of self-compatibility.

## Introduction

While a variety of mechanisms to promote outcrossing for diversification have evolved in angiosperms, selfing may nevertheless be favored in terms of reproductive assurance and inherent genetic transmission when these advantages outweigh a reduction in fitness (Darwin, 1876; Charlesworth and Vekemans, 2005; Franklin-Tong, 2008). In this evolutionary process, the switch from outcrossing to selfing mode is associated with the loss of self-incompatibility (SI) in many plant lineages (Stebbins, 1974; Goodwillie et al., 2005). SI is defined as the inability of a fertile hermaphrodite plant to produce zygotes after self-pollination, consequently promoting outcrossing, and diverse SI systems have evolved in angiosperms (de Nettancourt, 2001; Watanabe et al., 2012). The molecular basis of SI has been extensively studied in the family Brassicaceae, and it has been found to be controlled by a single highly polymorphic locus, the *S*-locus (Bateman, 1955; Watanabe et al., 2012), which comprises the male and female SI specificity genes *SP11* (*S*-locus protein 11)/*SCR* (*S*-locus cysteine rich protein) and *SRK* (*S*-locus receptor kinase), respectively (Suzuki et al., 1999; Schopfer et al., 1999; Takayama et al., 2000; Takasaki et al., 2000; Shiba et al., 2001). SP11/SCR is a small cysteine-rich protein localized in the pollen coat that acts as a ligand, and SRK is a transmembrane serine/threonine receptor kinase on the stigma epidermis that functions as a receptor for SP11/SCR. Their physical interaction, in an allele-specific manner, initiates the subsequent incompatible reaction to reject self pollen (Takayama et al., 2001), through an as yet inadequately understood downstream system.

The model plant *Arabidopsis thaliana* is a self-compatible, predominantly selfing species of Brassicaceae, whereas its ancestral state is thought to have been an obligate outcrosser enforced by SI (Kusaba et al., 2001; Bechsgaard et al., 2006; Tang et al., 2007; Shimizu et al., 2008) through haplotype-specific interaction of *SCR* and *SRK*. Functional orthologues of *SCR* and *SRK* are found in *A. lyrata* and *A. halleri*, SI congeners, as shown by the successful recovery of SI in *A. thaliana* in heterologous experiments with introduction of the orthologues (Nasrallah et al., 2002; Nasrallah et al., 2004; Tsuchimatsu et al., 2010; Fujii et al., 2020). Although such experiments provided the insight that *A. thaliana* genomic and physiological background is compatible with the orthologues in recovering SI, heterologous experiments might not have captured the functional context of nucleotide variations in *SCR* and/or *SRK*. For example, *A. lyrata* *SCR* and *SRK* were of different haplotype origin (Nasrallah et al., 2002; Nasrallah et al., 2004) and the transgenic *SCR* construct was driven by the non-native anther-specific promoter (Tsuchimatsu et al., 2010), which might not align with the attempt to reverse the evolutionary course that occurred in this locus in *A. thaliana*. Indeed, even within *A. thaliana* the evolution of SI has been diverse with different paths leading to disruption of *SCR* and/or *SRK* (Kusaba et al., 2001; Sherman-Broyles et al., 2007; Shimizu et al., 2008; Tsuchimatsu et al., 2017; Fujii et al., 2020).

In this study our aim is to understand the functional and evolutionary contexts of the loss of SI in *A. thaliana* by segregating the primary and secondary mutations. For this we compared functional and disrupted *SCRs* between *A. halleri* and *A. thaliana*, including their transcriptional regulation, from the same haplotype origin and elucidated the historical transition in *A. thaliana*.

## Materials and Methods

### Plant Material

*A. thaliana* ecotype Oldenburg (Old-1) was obtained from the Arabidopsis Biological Resource Center (ABRC; http://abrc.osu.edu/). Old-1 plants were grown in a growth chamber (BIOTRON LH-240S, NK system, Osaka, Japan) at 22°C under 8 h light/16 h dark photoperiod for the vegetative stage and 16 h light/8 h dark photoperiod for the reproductive stage. *A. halleri* Tada-mine accession (W302) was originally collected in Japan and developed by self-fertilization for five generations. W302 plants were grown in a growth chamber at 22°C under 16 h light/8 h dark photoperiod.

### Pollination Assay

Flower buds of Old-1 and W302 were emasculated at developmental stage 12 (Smyth et al., 1990) and incubated at 22°C on 1% agar medium until appropriate developmental stages. Each emasculated pistil at developmental stages 12, 13, 14, and 15 of W302 was self-pollinated. In Old-1, each emasculated pistil at developmental stages 12, 13, 14, and 15 was pollinated with pollen of W302 to test the female SI function of Old-1. After fixation and staining with aniline blue solution, pollen tubes were observed using fluorescence microscopy (Axio Imager A2, Carl Zeiss, Jena, Germany) according to Nou et al. (1991) and Watanabe et al. (1992).

### Determination of Full Structure of *SCR-A*

Genomic DNA and mRNA were extracted from Old-1 and W302 using Plant DNeasy and RNeasy kits (Qiagen, Hilden, Germany). First-strand cDNAs of anther and stigma were synthesized using a High Capacity RNA-to-cDNA Kit (Thermo Fisher Scientific, Waltham, MA, USA). The promoter and gene regions of *SCR-A* of Old-1 and W302 were amplified by genomic PCR (all primer information is summarized in **Supplementary Table S1**), subcloned into pCR2.1-TOPO vector (Thermo Fisher Scientific) and sequenced by an Applied Biosystems 3130 Genetic Analyzer (Thermo Fisher Scientific) according to Watanabe et al. (1994). The 5′- and 3′-UTRs of *SCR-A* were determined by First Choice RLM-RACE kit (Thermo Fisher Scientific), using anther cDNA of W302 based on Kakita et al. (2007).

### Quantification of mRNA Expression of *SCR-A* and *SRK-A*

Expression levels of *SCR-A* and *SRK-A* in *A. thaliana* and *A. halleri* were quantified by qRT-PCR, using anther and stigma cDNAs of Old-1 and W302, respectively, by Step One Realtime PCR System (Thermo Fisher Scientific) according to Osaka et al. (2013). 18S rRNA was used as an internal control.

### Transformation of *A. thaliana*

A 3,523-bp *AhSCR-A* fragment, consisting of 1,845-bp *AhSCR* promoter and 1,678-bp *AhSCR* gene, was amplified by genomic PCR and subcloned into pBI121 vector. Artificial chimeric *SCR-A* constructs, with one of the three major mutations of *AtSCR*, promoter fragments and former/latter parts of *SCR-A* coding region, were amplified from Old-1 and W302 by PCR with specific primers (**Supplementary Table S1**), respectively, connected and subcloned into pBI121 vector. Each vector was introduced into *Agrobacterium tumefaciens* strain GV3101 and transformed into Old-1 plants by the floral dip method according to Park et al. (2010).

## Results and Discussion

### Genetic and Functional Characteristics of *SCR* in *A. thaliana* and *A. halleri*

Thus far, the remnants of three haplotypes (haplogroups) at the *S*-locus have been identified in *A. thaliana*: haplogroup A is distributed predominantly in the northern hemisphere, haplogroup B is restricted to African islands, and haplogroup C is distributed mainly in Asia (Sherman-Broyles et al., 2007; Tang et al., 2007; Shimizu et al., 2008). In haplogroup A, disruption patterns in the *S*-locus are further classified into five variants, termed A-t1 to A-t5. While A-t2 and A-t3 groups have deleted the corresponding *SCR* genomic region, A-t1 and A-t5 still possess the *SCR* pseudogene (also known as Ψ*SCR1*) together with the *SRK* pseudogene and A-t4 has the *SCR* pseudogene with a functional *SRK*, reflecting the complex evolutionary history of selfing in *A. thaliana*.

**Figure 1:**
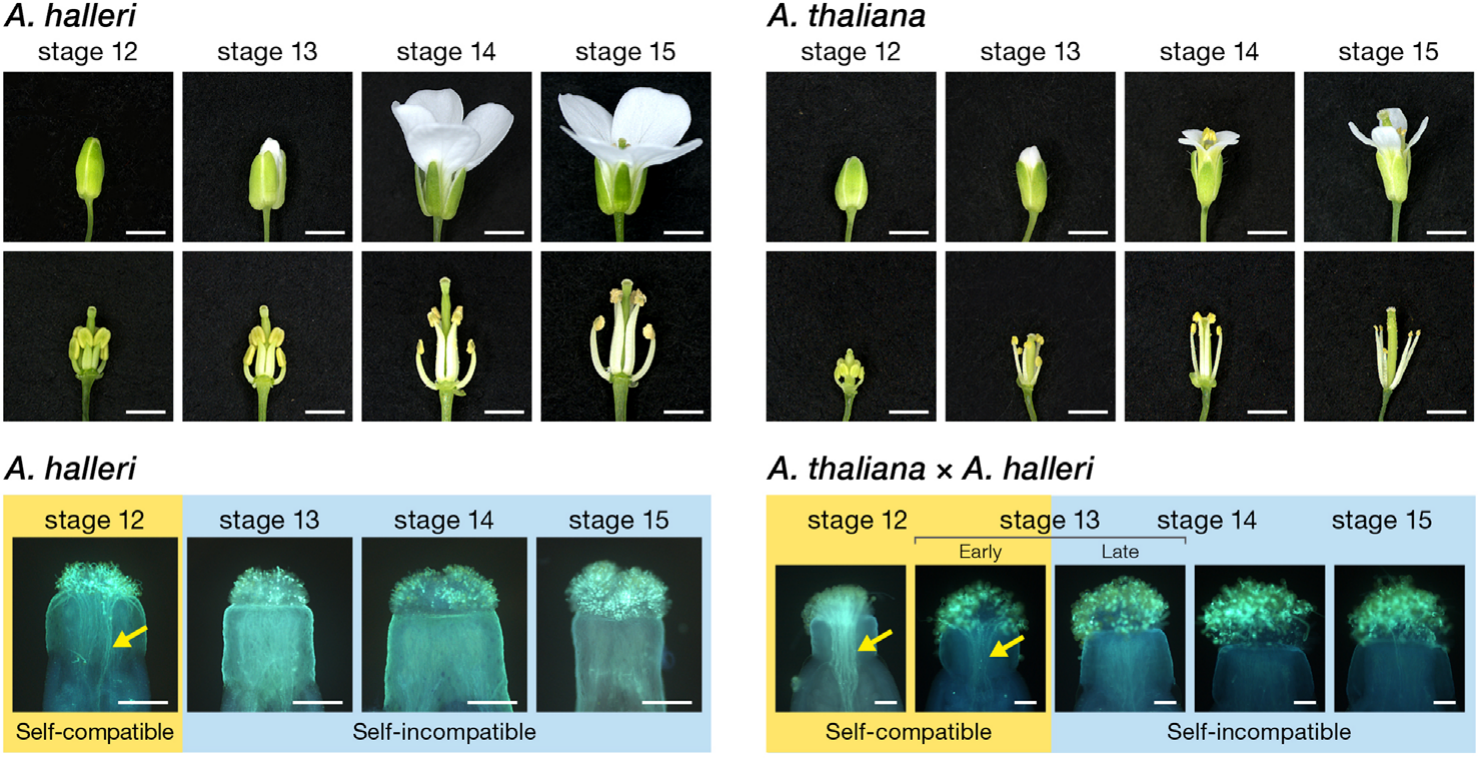
Floral morphology and pollination behaviour during flower development in *A. thaliana* and *A. halleri*. Appearance and inner morphological structure of flower buds, with floral developmental stages (Smyth et al., 1990). Flower buds of Old-1 and W302 were emasculated at developmental stage 12 and incubated at 22°C on 1% agar medium until appropriate developmental stages. Each emasculated pistil at developmental stages 12, 13, 14, and 15 was self-pollinated in W302 and cross-pollinated with pollen of W302 in Old-1 to test the female SI function of Old-1. Scale bar, 1 mm. Self-incompatible and self-compatible floral stages are highlighted with blue and yellow boarders, respectively, in self-pollinated pistils of *A. halleri* and *A. thaliana* pistils pollinated with *A. halleri* pollens. Arrows indicate growing pollen tubes. Scale bar, 0.1 mm.

**Figure 2:**
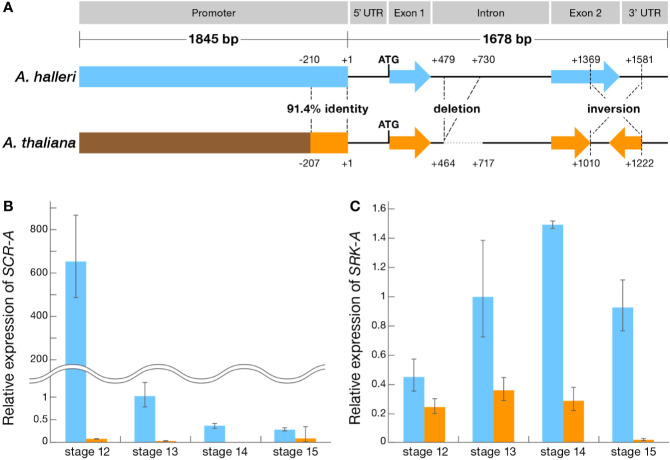
Genomic structure and expression profile of the SI genes in *A. thaliana* and *A. halleri*. **(A)** Schematic structure of *SCR-A* of *A. thaliana* and *A. halleri*. **(B)** Expression profile of *SCR-A* at flower developmental stages 12-15 in *A. thaliana* Old-1 (orange) and *A. halleri* W302 (blue). **(C)** Expression profile of *SRK-A* at flower developmental stages 12–15 in *A. thaliana* Old-1 and *A. halleri* W302. **(B, C)** Relative gene expression was determined by qRT-PCR and expression levels of transcripts at each stage are shown relative to *A. halleri* W302 at stage 13 (set to value of 1). Data from 4 biological replicates are shown. Error bars indicate ± SD.

Previously we reported the pivotal role of the 213-bp inversion in the coding region of *A. thaliana* *SCR*, in which SI can be restored by reverting the inversion (Tsuchimatsu et al., 2010). In order to segregate the functional context of the numerous mutation that may be responsible for disruption (i.e. primary mutation) or not directly related (i.e. secondary mutation) in both coding and upstream regulatory regions, we used a comparative panel of self-compatible *A. thaliana* Oldenburg accession (Old-1) and outcrosser *A. halleri* Tada-mine accession (W302). This panel enables the comparative functional dissection of the *S*-locus in Old-1 and W302 because they have the identical *S*-haplotype, haplogroup A, *trans*-specifically, and Old-1 still retains the female SI function with an intact *SRK-A* coding region while the male SI function is disrupted (Tsuchimatsu et al., 2010). These two lines also have an identical flower developmental process and pollination behavior (**Figure 1**). The structure of *SCR-A* in W302 consists of two exons (67-bp exon 1 and 188-bp exon 2) separated by an intron of 1157 bp with 52-bp 5′ and 214-bp 3′ UTRs, and a total length of 1,678 bp (**Figure 2A** and **Supplementary Figure S1**). In comparison to the *SCR-A* of W302, as we reported previously (Tsuchimatsu et al., 2010), the nonfunctional form of *SCR-A* in Old-1 has variations including a 252-bp deletion in the intron, minor indels (≤36 bp) in the exons and a 213-bp inversion in exon 2 (**Figure 2A** and **Supplementary Figure S1**). Compared to the relatively well conserved *SCR-A* coding sequences, the upstream element has extensive alterations; there is no overall homology between upstream sequences of Old-1 and W302, except for regions -1 to -210 (**Figure 2A** and **Supplementary Figure S1**). Thus, to summarize, there are three major differences in *SCR-A* between Old-1 and W302: limited homology in the promoter, a 252-bp deletion in the intron and a 213-bp inversion in exon 2.

To address the consequences of extensive alteration in upstream sequences, we compared expression of *SCR-A* between Old-1 and W302 in anthers during flower development (Smyth et al., 1990). Real-time quantitative expression analysis revealed a significantly lower level of *SCR*-*A* expression in Old-1 compared to W302 (**Figure 2B**). The overall temporal patterns were similar in both Old-1 and W302, with peak expression observed prior to anthesis (i.e. stage 12) followed by a drastic reduction in later stages (i.e. stages 13–15). This indicates that the native *SCR-A* promoter in Old-1 seems to be attenuated in achieving the “optimal” expression level as seen in W302. From analysis of expression of the female-specificity gene *SRK-A*, again a lower expression in Old-1 stigmas was observed at stage 12 (**Figure 2C**). However, the degree of reduction was only about 10 times for *SRK-A*, unlike *SCR-A* which showed an ~6500 times reduction. Taken together, these results indicate that the *S*-locus transcriptional level in Old-1 was strongly silenced, especially in *SCR-A* and to a lesser extent in *SRK-A*, which may constitute a critical step in the loss of SI in *A. thaliana*.

### Complete Experimental Reversal of the Historical Transition From Outcrossing to Selfing in *A. thaliana*

**Figure 3:**
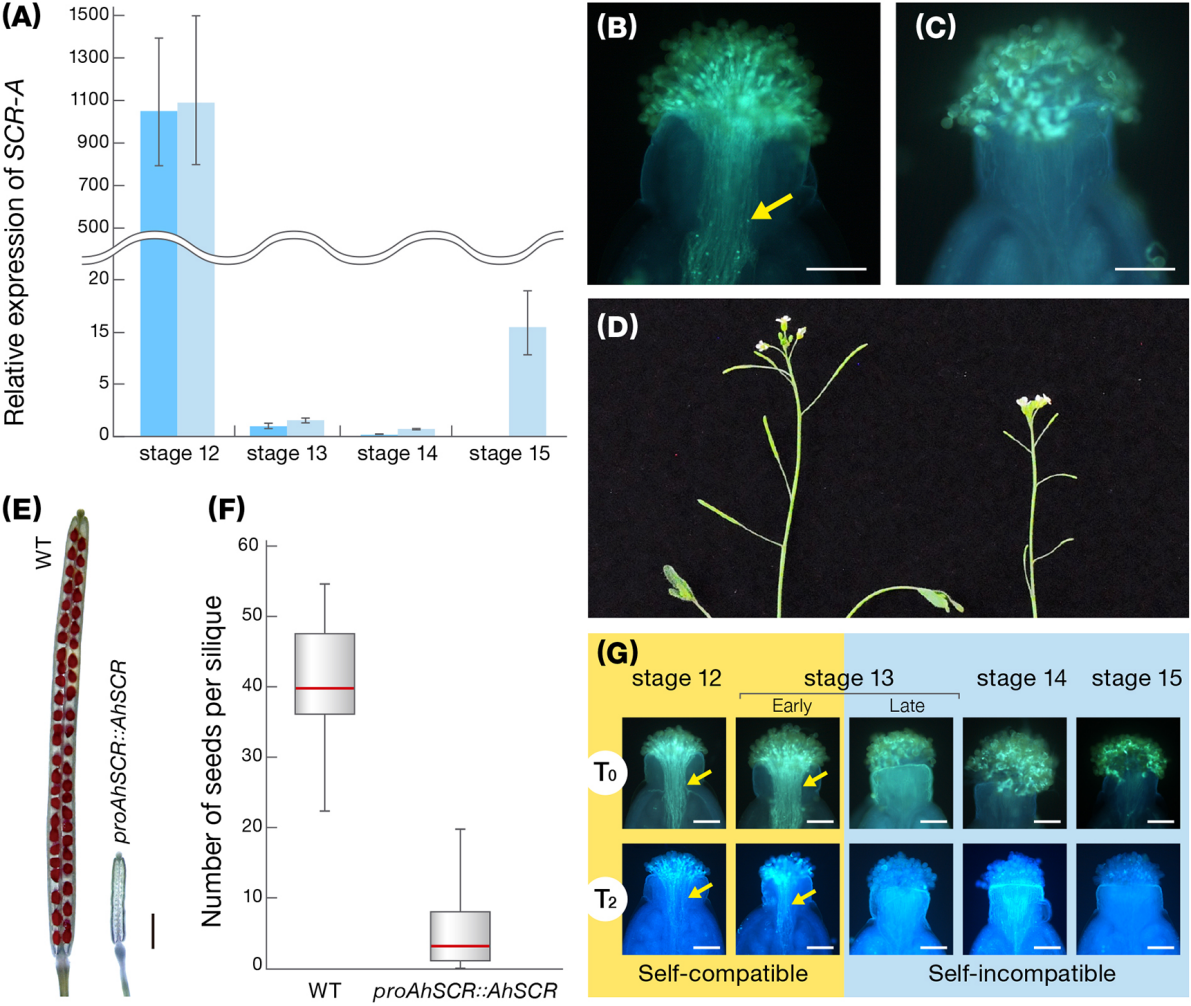
Characteristics of transgenic *A. thaliana* with a functional *SCR-A*. **(A)** Expression of *SCR-A* at flower developmental stages 12–15 in *proAhSCR::AhSCR* transgenic *A. thaliana* plants (light blue) and *A. halleri* W302 plants (blue). Relative gene expression was determined by qRT-PCR and expression levels of transcripts at each stage are shown relative to *A. halleri* W302 at stage 13 (set to value of 1). Data from 4 biological replicates are shown. Error bars indicate ± SD. **(B)** Pollen tubes accepted in the stigma of a selfed pistil of wild-type *A. thaliana* Old-1. Arrow indicates growing pollen tubes. Scale bar, 0.1 mm. **(C)** Pollen tubes inhibited on the stigma of a selfed pistil of *proAhSCR::AhSCR* transgenic *A. thaliana* plant. Scale bar, 0.1 mm. **(D)** Inflorescences of *proAhSCR::AhSCR* transgenic *A. thaliana* plant (right) and wild-type *A. thaliana* Old-1 (left). **(E)** Silique resulting from selfing of *proAhSCR::AhSCR* transgenic *A. thaliana* (right) and wild-type *A. thaliana* Old-1 (left). Scale bar, 1 mm. **(F)** Comparison of number of seeds per silique resulting from selfing of the *proAhSCR::AhSCR* transgenic *A. thaliana* plant and wild-type *A. thaliana* Old-1. Thirty siliques were examined from each plant. Red bars, gray boxes, and black whiskers represent the median, the interquartile range, and 1.5 times extension of the interquartile range, respectively. **(G)** Pollination phenotype in T_0_ and progeny plants (T_2_ shown as a representative of T_2_, T_3_, and T_6_ plants) of *proAhSCR::AhSCR* transgenic *A. thaliana*, with flower development stage. Arrows indicate growing pollen tubes. Scale bar, 0.1 mm.

Given that our representative lines of *A. halleri* (functional SI) and *A. thaliana* (disrupted SI) have an identical *S*-haplotype with three major mutations in the *SCR-A* of *A. thaliana* Old-1 (*AtSCR*), namely a promoter with limited homology, a 252-bp deletion in the intron and a 213-bp inversion in exon 2, we regarded *SCR-A* of *A. halleri* W302 (*AhSCR*) as the functional, original form of *AtSCR*. The 3,523-bp *AhSCR-A* fragment (designated *proAhSCR::AhSCR*), consisting of a 1,845-bp *AhSCR* promoter and 1,678-bp *AhSCR* coding region, was introduced into Old-1. Expression of *SCR-A* in transgenic *proAhSCR::AhSCR* Old-1 plants was similar to that in W302, and transgenic Old-1 plants rejected self pollen on their stigmas, resulting in siliques with no or very few seeds (**Figures 3A–F**). In T_2_, T_3_, and T_6_ plants with *proAhSCR::AhSCR*, which were produced through bud self-pollination, developmentally stable SI was observed during autopollination at stages 12 to 15 (**Figure 3G**), while previously we observed that the SI reaction on the female side was attenuated at a relatively late stage (Tsuchimatsu et al., 2010). This indicates that the SI trait was restored in these transgenic Old-1 plants and this was heritable. Together with our previous results (Tsuchimatsu et al., 2010), this demonstrates a complete experimental reversal of the historical transition from outcrossing to selfing in *A. thaliana*, and is basically consistent with the heterologous SI in *A. thaliana* (Nasrallah et al., 2002; Nasrallah et al., 2004). This finding also indicates that inactivation of the *S*-locus was a primary step in the evolution of selfing in *A. thaliana* (Nasrallah et al., 2002; Sherman-Broyles et al., 2007; Boggs et al., 2009).

### Discrimination of the Primary Inactivating Mutation and Decays for the Evolutionary Loss of SI in *A. thaliana*

**Figure 4:**
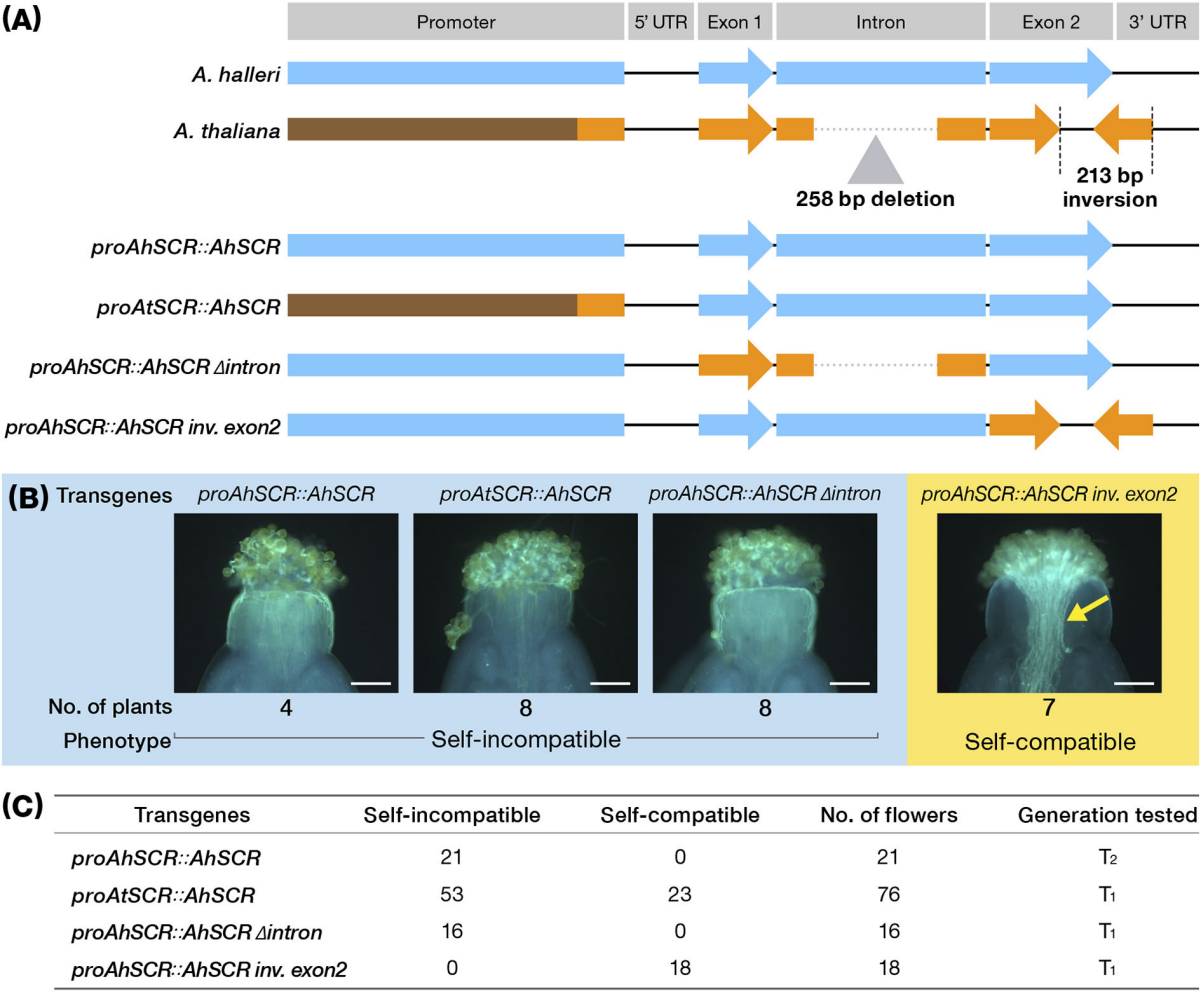
Schematic structure of artificial chimeric *SCR-A* and their effect on pollination in transgenic *Arabidopsis* plants. **(A)** Schematic structure of chimeric *SCR-A*. **(B)** Pollen tube behaviour on selfed pistils in each transgenic plant. Arrow indicates growing pollen tubes in the pistil. Scale bar, 0.1 mm. **(C)** Number of self-incompatible/self-compatible flowers in T_1_ or T_2_ generation of each transgenic plant.

To examine the functional consequences of the three *SCR-A* mutations in *A. thaliana* Old-1 (that is promoter alteration, deletion in the intron and inversion in exon 2), we conducted transgenic experiments in which chimeric constructs were introduced into Old-1 (**Figure 4**). As explained above, introduction of a W302-type *SCR-A* promoter and coding region (*proAhSCR::AhSCR*) achieved complete recovery of SI in all flowers as expected. However, to our surprise, the Old-1 promoter with W302 *SCR-A* coding region (designated *proAtSCR::AhSCR*) showed SI in 53 out of 76 flowers (**Figures 4A, C**), indicating the *SCR-A* promoter of Old-1 is partially functional in inducing transcription of *SCR-A*. The W302 promoter and coding sequences combined with the Old-1 deletion in the intron (designated *proAhSCR::AhSCR Δintron*) conferred SI in all flowers. The SI reaction was strongly prohibited by inversion of exon 2 regardless of the W302 promoter and exon 1 and intron (designated *proAhSCR::AhSCR inv. exon2*). This is consistent with the role of the inversion in exon 2 as the primary and strongest determining factor in the evolution of SI reaction, whereas the other two mutations of the deleted intron and attenuated expression are the result of decay. Hence, we conclude that the primary inactivating mutation for the evolutionary loss of SI in *A. thaliana* is the exon 2-disrupting inversion in *SCR-A*, which was a primary and definitive evolutionary event for the transition from outcrossing to selfing in *A. thaliana*.

### Functional and Evolutionary Context of Transitional History in *A. thaliana*

**Figure 5:**
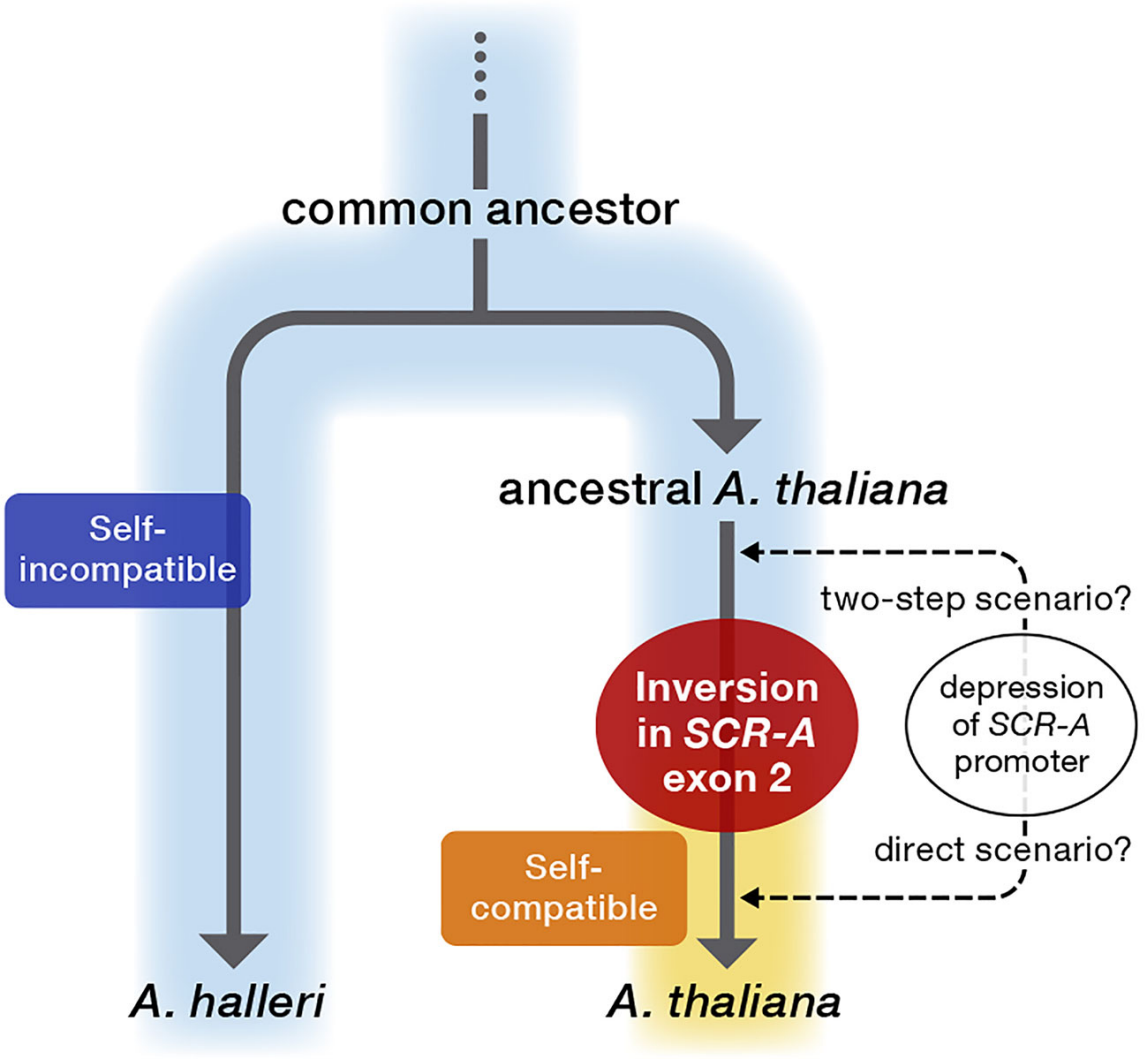
Proposed model of the evolutionary process of loss of self-incompatibility in genus *Arabidopsis*. After an evolutionary split from the common ancestor, ancestral *A. thaliana* still retained SI, as in the sister SI species *A. halleri*. The inactivating mutation resulting in the evolutionary loss of SI in *A. thaliana* occurred as an exon 2-disrupting inversion in *SCR-A*, which was a primary and definitive evolutionary event for the transition from outcrossing to selfing in *A. thaliana*. In addition, the *SCR-A* promoter was attenuated in this process, and thus two scenarios can be proposed for the evolutionary loss of SI in *A. thaliana*: 1) a two-step scenario in which *A. thaliana* ﬁrst evolved partial SI by depression of *proAtSCR* and then complete SC by the inversion in *SCR-A* exon 2, and 2) a direct scenario in which *A. thaliana* lost SI by the inversion in *SCR-A* exon 2 together with a depression of *proAtSCR* as a secondary decay.

The three major mutations in *SCR-A* are nearly fixed in a range of European *A. thaliana* accessions (**Supplementary Figure S2**), suggesting that these mutations in *SCR-A* had already occurred before differentiation of these accessions. Coupled with the prediction of a relatively recent origin of self-compatibility in *A. thaliana* (Nasrallah et al., 2002; Bechsgaard et al., 2006; Shimizu et al., 2008; Shimizu and Tsuchimatsu, 2015), it is conceivable that the ancestor of *A. thaliana* still retained SI after the split from the common ancestor of the sister species *A. halleri* and *A. lyrata* (Koch et al., 2000), in which all SI components were under selective constraint for the maintenance of SI. Interestingly, *proAtSCR::AhSCR* transgenic plants showed weakening of SI in some flowers, indicating the expression level of *AhSCR* driven by *proAtSCR* was on the threshold of the level required for an incompatibility reaction, resulting in partial SI (Shimizu and Tsuchimatsu, 2015) in *proAtSCR::AhSCR* transgenic plants. The partial recovery of SI could be attributed to two scenarios for the evolutionary loss of SI in *A. thaliana* (**Figure 5**): 1) a two-step scenario in which *A. thaliana* ﬁrst evolved partial SI by depression of *proAtSCR* and then complete SC by inversion in *SCR-A* exon 2 (Castric et al., 2014; Shimizu and Tsuchimatsu, 2015), and 2) a direct scenario in which *A. thaliana* lost SI by inversion in *SCR-A* exon 2 together with a depression of *proAtSCR* as a secondary decay (Tsuchimatsu et al., 2010). Due to limitations in the methodology of calculating molecular clocks for large structural alterations compared to small mutations, it is not possible to reliably determine whether depression of *proAtSCR* proceeded the inversion or not. However, in either scenario, *SCR-A* provides the molecular basis of transition from outcrossing to selfing, leading to accumulation of mutations and decays following release from selective constraints.

## Conclusion

Our work has established the functional and evolutionary contexts of the major mutations in promoters, exons and introns of *SCR* and has suggested a transcriptional contribution to achieve the complete switch from outcrossing to selfing in *A. thaliana*. It is notable that the functionality of the pseudogene promoter has been retained. The findings from analysis of the fixed mutations in *SCR-A* further support the hypotheses of a recent origin and selection of selfing in *A. thaliana* (Bechsgaard et al., 2006; Shimizu et al., 2008) and the advantage of mutation in the male specificity gene (Tsuchimatsu et al., 2010), rather than the female specificity gene, for the evolution of self-compatibility (Uyenoyama et al., 2001; Busch and Schoen, 2008; Tsuchimatsu and Shimizu, 2013).

## Data Availability Statement

The datasets presented in this study can be found in online repositories. The names of the repository/repositories and accession number(s) can be found in the article/supplementary material.

## Author Contributions

KS, TT, KKS, ST, GS, and MW planned and designed the study, and wrote the paper. KN, EAW, CH, TO, MT, HM-S, and YK performed sequencing, expression profiling, crossing experiment and transgenic analysis. AK conducted nucleotide sequence analysis, and wrote the paper. KY conducted nucleotide sequence analysis. KS, KN, and EAW contributed equally to the study. All authors contributed to the article and approved the submitted version.

## Funding

This work was supported in part by MEXT KAKENHI (Grant Numbers 16H06467 to ST; 16H06469 to KKS; 17H05833, 19H04851 to TT; 19H04870 to KY; 16H06470, 16H06464, and 16K21727 to MW), JSPS KAKENHI (Grant Numbers 16H06380 to ST; 20K05982 to GS; 20H02956 to KS; 17H00821, 18KT0048, 19K22342 to MW) and JSPS Bilateral Programs (Grant Number 18032211-000481 to MW), by the Swiss National Science Foundation (31003A_159767 to KKS) and by the Research Funding for Computational Software Supporting Program from Meiji University to KY.

## Conflict of Interest

The authors declare that the research was conducted in the absence of any commercial or financial relationships that could be construed as a potential conflict of interest.

## Supplementary Material

Supplementary Figure 1 Nucleotide sequence comparison of *SCR-A* between *A. thaliana* and *A. halleri*. Promoter and coding region of *SCR-A* aligned between *A. thaliana* and *A. halleri*. Identical nucleotide sequences between *A. thaliana* and *A. halleri* are highlighted with black background. In promoter region, open box outlined by red lines indicates a homologous region between *A. thaliana* and *A. halleri*. In coding region, two exons, the deletion in intron and the inversion in exon 2 are highlighted with red, yellow and blue backgrounds, respectively. Black arrows indicate the primer site for qRT-PCR.

Supplementary Figure 2 Alignment of nucleotide sequences of *SCR-A* among *A. thaliana* accessions. Promoter and coding region of *SCR-A* aligned among 11 A*. thaliana* accessions in haplogroup A. Identical conserved sequences are highlighted with black background.

Supplementary Table 1 List of Primers
