# Supplementary material for: Double-Locking Mechanism of Self-Compatibility in Arabidopsis thaliana: The Synergistic Effect of Transcriptional Depression and Disruption of Coding Region in the Male Specificity Gene
Source: Front Plant Sci. 2020 Sep 11;11:576140. doi: 10.3389/fpls.2020.576140 (PMC7517786; doi:10.3389/fpls.2020.576140)
Supplement: Supplementary Figure 1 — Nucleotide sequence comparison of SCR-A between A. thaliana and A. halleri. Promoter and coding region of SCR-A aligned between A. thaliana and A. halleri. Identical nucleotide sequences between A. thaliana and A. halleri are highlighted with black background. In promoter region, open box outlined by red lines indicates a homologous region between A. thaliana and A. halleri. In coding region, two exons, the deletion in intron and the inversion in exon 2 are highlighted with red, yellow and blue backgrounds, respectively. Black arrows indicate the primer site for qRT-PCR. [file DataSheet_2.pdf]

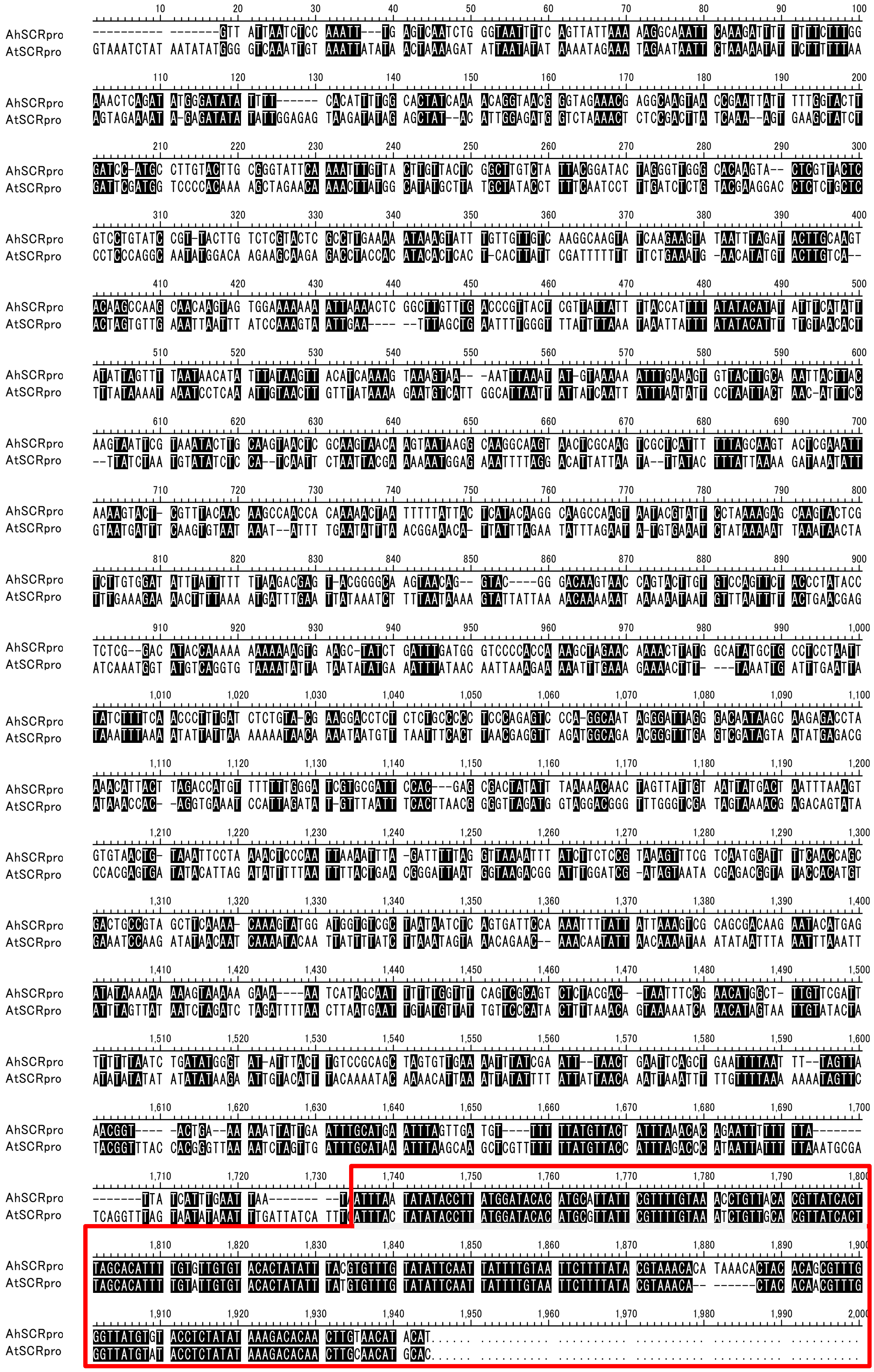

Promoter region of *SCR-A*

Supplementary Figure S1
(Figure continues on next page)

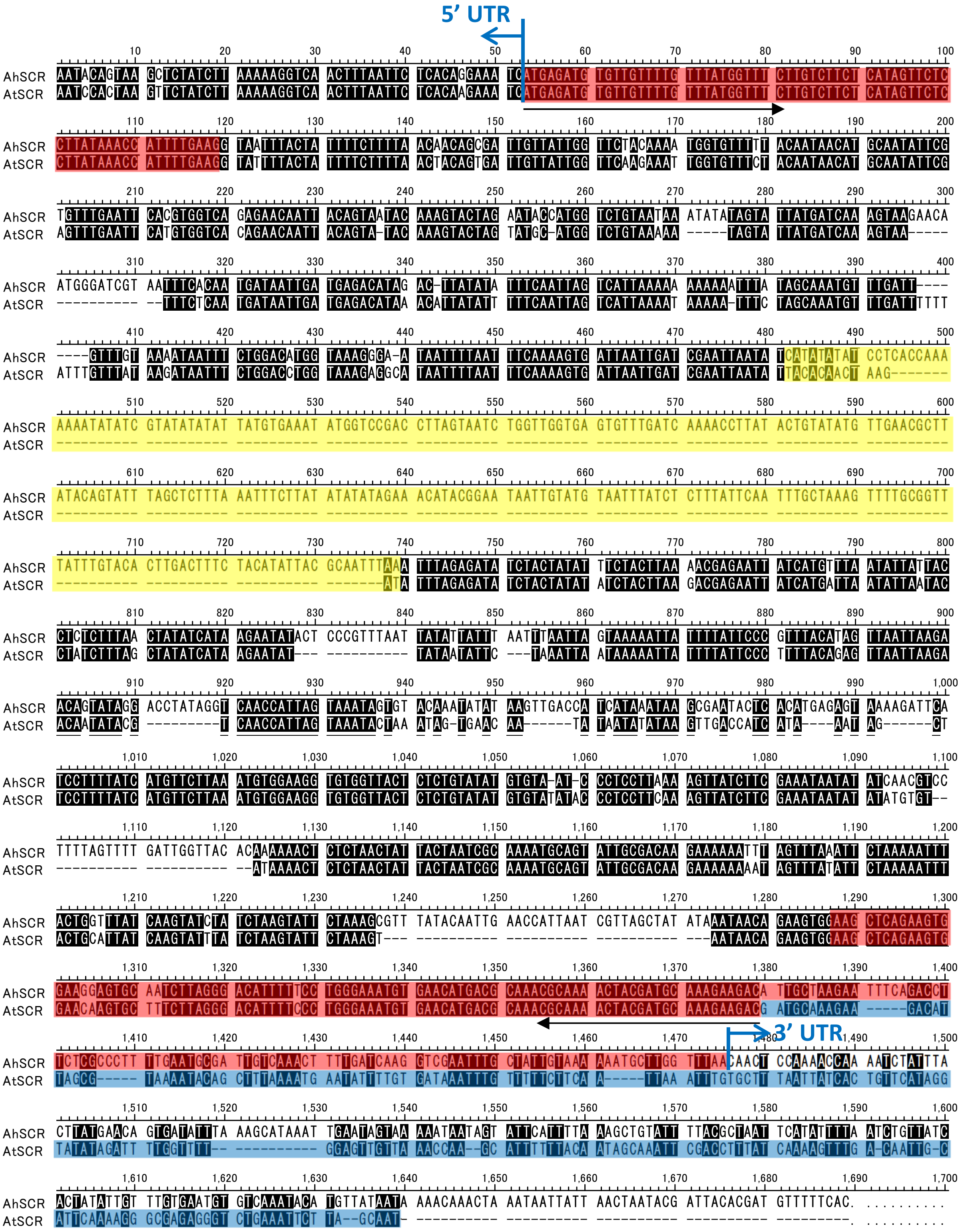

Coding region of *SCR-A*

Nucleotide sequence comparison of *SCR-A* between *A. thaliana* and *A. halleri.*

Supplementary Figure S1 (continued)
