# Supplementary material for: Double-Locking Mechanism of Self-Compatibility in Arabidopsis thaliana: The Synergistic Effect of Transcriptional Depression and Disruption of Coding Region in the Male Specificity Gene
Source: Front Plant Sci. 2020 Sep 11;11:576140. doi: 10.3389/fpls.2020.576140 (PMC7517786; doi:10.3389/fpls.2020.576140)
Supplement: Supplementary Figure 2 — Alignment of nucleotide sequences of SCR-A among A. thaliana accessions. Promoter and coding region of SCR-A aligned among 11 A. thaliana accessions in haplogroup A. Identical conserved sequences are highlighted with black background. [file DataSheet_3.pdf]

```
                  10        20        30        40        50        60        70        80        90       100
          ----|----|----|----|----|----|----|----|----|----|----|----|----|----|----|----|----|----|----|----|
Col-0     GATATTAATATATAAAATAGAAATAGAATAATTCTAAAAATATTCTTTTTTAAAGTAGAAAATAGAGATATATATTGGAGAGTAAGATATAGAGCTATAC 100
C24       GATATTAATATATAAAATAGAAATAGAATAATTCTAAAAATATTCTTTTTTAAAGTAGAAAATAGAGATATATATTGGAGAGTAAGATATAGAGCTATAC 100
Ca-0      GATATAAATATATAAAATAGAAATAGAATAATTCTAAAAATATTCTTTTTTAAAGTAGAAAATAGAGATATACATTGGAGAGTAAGATATAGAGCTATAC 100
Gie-0     GATATTAATATATAAAATAGAAATAGAATAATTCTAAAAATATTCTTTTTTAAAGTAGAAAATAGAGATATACATTGGAGAGTAAGATATAGAGCTATAC 100
Old-1     GATATTAATATATAAAATAGAAATAGAATAATTCTAAAAATATTCTTTTTTAAAGTAGAAAATAGAGATATACATTGGAGAGTAAGATATAGAGCTATAC 100
Pog-0     -------------------------------TTCTAAAAATATTCTTTTTTAAAGTAGAAAATAGAGATATACATTGGAGAATAAGATATAGAGCTATAC 69
Ws-0      GATATTAATATATAAAATAGAAATAGAATAATTCTAAAAATATTCTTTTTTAAAGTAGAAAATAGAGATATATATTGGAGAGTAAGATATAGAGCTATAC 100
Da-1-12   GATATTAATATATAAAATAGAAATAGAATAATTCTAAAAATATTCTTTTTTAAAGTAGAAAATAGAGATATACATTGGAGAGTAAGATATAGAGCTATAC 100
Mz-0      GATATTAATATATAAAATAGAAATAGAATAATTCTAAAAATATTCTTTTTTAAAGTAGAAAATAGAGATATATATTGGAGAGTAAGATATAGAGCTATAC 100
Wa-1      GATATTAATATATAAAATAGAAATAGAATAATTCTAAAAATATTCTTTTTTAAAGTAGAAAATAGAGATATACATTGGAGAGTAAGATATAGAGCTATAC 100
La-0      GATATTAATATATAAAATAGAAATAGAATAATTCTAAAAATATTCTTTTTTAAAGTAGAAAATAGAGATATACATTGGAGAGTAAGATATAGAGCTATAC 100

                 110       120       130       140       150       160       170       180       190       200
          ----|----|----|----|----|----|----|----|----|----|----|----|----|----|----|----|----|----|----|----|
Col-0     ATTGGAGATGGTCTAAAACTCTCCGACTTATCAAAAGTGAAGCTATCTGATTCGAT-GGTCCCCACAAAAGCTAGAACAAAACTTATGGCATATGCTTAT 199
C24       ATTGGAGATGGTCTAAAACTCTCCGACTTATCAAAAGTGAAGCTATCTGATTCGAT-GGTCCCCACAAAAGCTAGAACAAAACTTATGGCATATGCTTAT 199
Ca-0      ATTGGAGATGGTCTAAAGCTCTCCGACTTATCAAAAGTGAAGCTATCTGATTCGATGGGTCCCCACAAAAGCTAGAACAAAACTTATGGCATATGCTTAT 200
Gie-0     ATTGGAGATGGTCTAAAGCTCTCCGACTTATCAAAAGTGAAGCTATCTGATTCGATGGGTCCCCACAAAAGCTAGAACAAAACTTATGGCATATGCTTAT 200
Old-1     ATTGGAGATGGTCTAAAGCTCTCCGACTTATCAAAAGTGAAGCTATCTTATTCGAT-GGTCCCCACAAAAGCTAGAACAAAACTTATGGCATATGCTTAT 199
Pog-0     ATTGGAGATGGTCTAAAACTCTCCGACTTATCAAAAGTGAAGCTATCTGATTCGATGGGTCCCCACAAAAGCTAGAACAAAACTTATGGCATATGCTTAT 169
Ws-0      ATTGGAGATGGTCTAAAACTCTCCGACTTATCAAAAGTGAAGCTATCTGATTCGATGGGTCCCCACAAAAGCTAGAACAAAACTTATGGCATATGCTTAT 200
Da-1-12   ATTGGAGATGGTCTAAAACTCTCCGACTTATCAAAAGTGAAGCTATCTGATTCGATGGGTCCCCACAAAAGCTAGAACAAAACTTATGGCATATGCTTAT 200
Mz-0      ATTGGAGATGGTCTAAAACTCTCCGACTTATCAAAAGTGAAGCTATCTGATTCGATGGGTCCCCACAAAAGCTAGAACAAAACTTATGGCATATGCTTAT 200
Wa-1      ATTGGAGATGGTCTAAAGCTCTCCGACTTATCAAAAGTGAAGCTATCTGATTCGATGGGTCCCCACAAAAGCTAGAACAAAACTTATGGCATATGCTTAT 200
La-0      ATTGGAGATGGTCTAAAGCTCTCCGACTTATCAAAAGTGAAGCTATCTGATTCGATGGGTCCCCACAAAAGCTAGAACAAAACTTATGGCATATGCTTAT 200

                 210       220       230       240       250       260       270       280       290       300
          ----|----|----|----|----|----|----|----|----|----|----|----|----|----|----|----|----|----|----|----|
Col-0     GCTATACCTTTTCAATCCTTTGATCTCTGTACGAAGGACCTCTCTGCTCCCTCCCAGGCAATATGGACAAGAAGCAAGAGACCTACCACATACACTCACT 299
C24       GCTATACCTTTTCAATCCTTTGATCTCTGTACGAAGGACCTCTCTGCTCCCTCCCAGGCAATATGGACAAGAAGCAAGAGACCTACCACATACACTCACT 299
Ca-0      GCTATACCTTTTCAATCCTTTGATCTCTGTACGAAGGACCTCTCTGCTCCCTCCCAGGCAATATGGACAAGAAGCAAGAGACCTACCACATACACTCACT 300
Gie-0     GCTATACCTTTTCAATCCTTTGATCTCTGTACGAAGGACCTCTCTGCTCCCTCCCAGGCAATATGGACAAGAAGCAAGAGACCTACCACATACACTCACT 300
Old-1     GCTATACCTTTTCAATCCTTTGATCTCTGTACGAAGGACCTCTCTGCTCCCTCCCAGGCAATATGGACAAGAAGCAAGAGACCTACCACATACACTCACT 299
Pog-0     GCTATACCTTTTCAATCCTTTGATCTCTGTACGAAGGACCTCTCTGCTCCCTCCCAGGCAATATGGACAAGAAGCAAGAGACCTACCACATACACTCACT 269
Ws-0      GCTATACCTTTTCAATCCTTTGATCTCTGTACGAAGGACCTCTCTGCTCCCTCCCAGGCAATATGGACAAGAAGCAAGAGACCTACCACATACACTCACT 300
Da-1-12   GCTATACCTTTTCAATCCTTTGATCTCTGTACGAAGGACCTCTCTGCTCCCTCCCAGGCAATATGGACAAGAAGCAAGAGACCTACCACATACACTCACT 300
Mz-0      GCTATACCTTTTCAATCCTTTGATCTCTGTACGAAGGACCTCTCTGCTCCCTCCCAGGCAATATGGACAAGAAGCAAGAGACCTACCACATACACTCACT 300
Wa-1      GCTATACCTTTTCAATCCTTTGATCTCTGTACGAAGGACCTCTCTGCTCCCTCCCAGGCAATATGGACAAGAAGCAAGAGACCTACCACATACACTCACT 300
La-0      GCTATACCTTTTCAATCCTTTGATCTCTGTACGAAGGACCTCTCTGCTCCCTCCCAGGCAATATGGACAAGAAGCAAGAGACCTACCACATACACTCACT 300

                 310       320       330       340       350       360       370       380       390       400
          ----|----|----|----|----|----|----|----|----|----|----|----|----|----|----|----|----|----|----|----|
Col-0     CACTTATTCGATTTTTTTTTCTGAAATGAACATATGTACTTGTCAACTAGTGTTGAAATTAATTTATCCAAAGTAATTGAATTTAGCTGAATTTTGGGTT 399
C24       CACTTATTCGATTTTTTTTTCTGAAATGAACATATGTACTTGTCAACTAGTGTTGAAATTAATTTATCCAAAGTAATTGAATTTAGCTGAATTTTGGGTT 399
Ca-0      CACTTATTCGATTTTTTTTCCTGAAATGAACATATGTACTTGTCAACTAGTGTTGAAATTAATTTATCCAAAGTAATTGAATTTAGCTGAATTTTGGGTT 400
Gie-0     CACTTATTCGATTTTTTTTCCTGAAATGAACATATGTACTTGTCAACTAGTGTTGAAATTAATTTATCCAAAGTAATTGAATTTAGCTGAATTTTGGGTT 400
Old-1     CACTTATTCGATTTTTTTTCCTGAAATGAACATATGTACTTGTCAACTAGTGTTGAAATTAATTTATCCAAAGTAATTGAATTTAGCTGAATTTTGGGTT 399
Pog-0     CACTTATTCGATTTTTTTTCCTGAAATGAACATATGTACTTGTCAACTAGTGTTGAAATTAATTTATCCAAAGTAATTGAATTTAGCTGAATTTTGGGTT 369
Ws-0      CACTTATTCGATTTTTTTTTCTGAAATGAACATATGTACTTGTCAACTAGTGTTGAAATTAATTTATCCAAAGTAATTGAATTTAGCTGAATTTTGGGTT 400
Da-1-12   CACTTATTCGATTTTTTTTTCTGAAATGAACATATGTACTTGTCAACTAGTGTTGAAATTAATTTATCCAAAGTAATTGAATTTAGCTGAATTTTGGGTT 400
Mz-0      CACTTATTCGATTTTTTTTTCTGAAATGTACATATGTACTTGTCAACTAGTGTTGAAATTAATTTATCCAAAGTAATTGAATTTAGCTGAATTTTGGGTT 400
Wa-1      CACTTATTCGATTTTTTTTTCTGAAATGAACATATGTACTTGTCAACTAGTGTTGAAATTAATTTATCCAAAGTAATTGAATTTAGCTGAATTTTGGGTT 400
La-0      CACTTATTCGATTTTTTTTCCTGAAATGAACATATGTACTTGTCAACTAGTGTTGAAATTAATTTATCCAAAGTAATTGAATTTAGCTGAATTTTGGGTT 400

                 410       420       430       440       450       460       470       480       490       500
          ----|----|----|----|----|----|----|----|----|----|----|----|----|----|----|----|----|----|----|----|
Col-0     TATTTTAAATAAATTATTTATATACATTTTTGTAACACTTTTATAAAATAAATCCTCAAATTGTAACTTGTTTATAAAAGAATGTCATTGGCATTAATTA 499
C24       TA-TTTAAATAAATTATTTATATACATTTTTGTAACACTTTTATAAAATAAATCCTCAAATTGTAACTTGTTTATAAAAGAATGTCATTGGCATTAATTA 498
Ca-0      TATTTTAAATAAATTATTTATATACATTTTTGCAACACTTTTATAAAATAAATCCTCAAATTGTAACTTGTTTATAAAAGAATGTCATTGCCATTAATTA 500
Gie-0     TATTTTAAATAAAT-----------ATTTTTGTAACACTTTTATAAAATAAATCCTCAAATTGTAACTTGTTTATAAAAGAATGTCATTGCCATTAATTA 489
Old-1     TATTTTAAATAAATTATTTATATACATTTTTGCAACACTTTTATAAAATAAATCCTCAAATTGTAACTTGCTTATAAAAGAATGTCATTGCCATTAATTA 499
Pog-0     TATTTTAAATAAATTATTTATATACATTTTTGCAACACTTTTATAAAATAAATCCTCAAATTGTAACTTGTTTATAAAAGAATGTCATTGCCATTAATTA 469
Ws-0      TATTTTAAATAAATTATTTATATACATTTTTGTAACACTTTTATAAAATAAATCCT---------AACTTGTTTATAAAAGAATGTCATTGGCATTAATTA 492
Da-1-12   TATTTTAAATAAATTATTTATATACATTTTTGCAACACTTTTATAAAATAAATCCTCAAATTGTAACTTGTTTATAAAAGAATGTCATTGGCATTAATTA 500
Mz-0      TATTTTAAATAAATTATTTATATACATTTTTGCAACACTTTTATAAAATAAATCCTCAAATTGTATCTTGTTTATAAAAGAATGTCATTGGCATTAATTA 500
Wa-1      TATTTTAAATAAATTATTTATATACATTTTTGCAATACTTTTATAAAATAAATCCTCAAATTGTAACTTGTTTATAAAAGAATGTCATTGGCATTAATTA 500
La-0      TATTTTAAATAAATTATTTATATACATTTTTGCAACACTTTTATAAAATAAATCCTCAAATTGTAACTTGTTTATAAAAGAATGTCATTGGCATTAATTA 500
```

Supplementary Figure S2

(Figure continues on next page)

```
                 510       520       530       540       550       560       570       580       590       600
          ----|----|----|----|----|----|----|----|----|----|----|----|----|----|----|----|----|----|----|----|
Col-0     TTATCAATTATTTAATATTCCTAATTACTAACATTTCCTTATCTAATGTATATCTCCATCAATTCTAATTACGAAAAAATGGAGAAATTTTAGGACATTA 599
C24       TTATCAATTATTTAATATTCCTAATTACTAACATTTCCTTATCTAATGTATATCTCCATCAATTCTAATTACGAAAAAATGGAGAAATTTTAGGACATTA 598
Ca-0      TTATCAATTATTTAATATTCCTAATTACTAACATTTCCTTATCTAATGTATATCTCCATCAATTCTAATTACGAAAAAATGGAGAAATTTTAGGACATTA 600
Gie-0     TTATCAATTATTTAATATTCCTAATTACTAACATTTCCTTATCTAATGTATATCTCCATCAATTCTAATTACGAAAGAATGGAGAAATTTTAGGACATTA 589
Old-1     TTATCAATTATTTAATATTCCTAATTACTAACATTTCCTTATCTAATGTATATCTCCATCAATTCTAATTACGAAAAAATGGAGAAATTTTAGGACATTA 599
Pog-0     TTATCAATTATTTAATATTCCTAATTACTAACATTTTCTTATCTAATGTATATCTCCATCAATTCTAATTACGAAAAAATGGAGAAATTTTAGGACATTA 569
Ws-0      TTATCAATTATTTAATATTCCTAATTACTAACATTTCCTTATCTAATGTATATCTCCATCAATTCTAATTACGAAAAAATGGAGAAATTTTAGGACATTA 592
Da-1-12   TTATCAATTATTTAATATTCCTAATTACTAACATTTCCTTATCTAATGTATATCTCCATCAATTCTAATTACGAAAAAATGGAGAAATTTTAGGACATTA 600
Mz-0      TTATCAATTATTTAATATTCCTAATTACTAACATTTCGTTATCTAATGTATATCTCCATCAATTCTAATTACGAAAAAATGGAGAAATTTTAGGACATTA 600
Wa-1      TTATCAATTATTTAATATTCCTAATTACTAATATTTCCTTATCTAATGTATATCTCCATCAATTCTAATTACGAAAAAATGAAGAAATTTTAGGACATTA 600
La-0      TTATCAATTATTTAATATTCCTAATTACTAACATTTCCTTATCTAATGTATATCTCCATCAATTCTAATTACGAAAAAATGGAGAAATTTTAGGACATTA 600

                 610       620       630       640       650       660       670       680       690       700
          ----|----|----|----|----|----|----|----|----|----|----|----|----|----|----|----|----|----|----|----|
Col-0     TTAATATTATACTTTATTAAAAGATAAATATTGTAATGATTTCAAGTGTAATAAATATTTTGAATATTTAACGGAAACATTATTTAGAATATTTAGAATA 699
C24       TTAATATTATACTTTATTAAAAGATAAATATTGTAATGATTTCAAGTGTAATAAATATTTTGAATATTTAACGGAAACATTATTTAGAATATTTAGAATA 698
Ca-0      TTAATATTATACTTTATTAAAAGATAAATATTGTAATGATTTCAAGTGTAATAAATATTTTGAATATTTAACGGAAACATTATTTAGAATATTTAGAATA 700
Gie-0     TTAATATTATACTTTATTAAAAGATAAATATTGTAATGATTTCAAGTGTAATAAATATTTTGAATATTTAACGGAAACATTATTTAGAATATTTAGAATA 689
Old-1     TTAATATTATACTTTATTAAAAGATAAATATTGTAATGATTTCAAGTGTAATAAATATTTTGAATATTTAACGGAAACATTATTTAGAATATTTAGAATA 699
Pog-0     TTAATATTATACTTTATTAAAAGATAAATATTGTAATGATTTCAAGTGTAATAAATATTTTGAATATTTAACGGAAACATTATTTAGAATATTTAGAATA 669
Ws-0      TTAATATTATACTTTATTAAAAGATAAATATTGTAATGATTTCAAGTGTAATAAATATTTTGAATATTTAACGGAAACATTATTTAGAATATTTAGAATA 692
Da-1-12   TTAATATTATACTTTATTAAAAGATAAATATTGTAATGATTTTAAGTGTAATAAATATTTTGAATATTTAACGGAAACATTATTTAGAATATTTAGAATA 700
Mz-0      TTAATATTATACTTTATTAAAAGATAAATATTGTAATGATTTCAAGTGTAATAAATATTTTGAATATTTAACGGAAACATTATTTAGAATATTTAGAATA 700
Wa-1      TTAATATTATACTTTATTAAAAGATAAATATTGTAATGATTTCAAGTGTAATAAATATTTTGAATATTTAACGGAAACATTATTTAGAATATTTAGAATA 700
La-0      TTAATATTATACTTTATTAAAAGATAAATATTGTAATGATTTCAAGTGTAATAAATATTTTGAATATTTAACGGAAACATTATTTAGAATATTTAGAATA 700

                 710       720       730       740       750       760       770       780       790       800
          ----|----|----|----|----|----|----|----|----|----|----|----|----|----|----|----|----|----|----|----|
Col-0     TGTGAAATCTATAAAAATTAAATAACTATTTGAAAGAAAACTTTTAAAATGATTTGAATTATAAATCTTTTAATAAAAGTATTATTAAAACAAAAAAATAA 799
C24       TGTGAAATCTATAAAAATTAAATAACTATTTGAAAGAAAACTTTTAAAATGATTTGAATTATAAATCTTTTAATAAAAGTATTATTAAAACAAAAAAATAA 798
Ca-0      TGTGAAATCTATAAAAATTAAATAACTATTTGAAAGAAAACTTTTAAAATGATTTGAATTATAAATCTTTTAATAAAAGTATTATTAAAACAAAAAAATAA 800
Gie-0     TGTGAAATCTATAAAAATTAAATAACTATTTGAAAGAAAACTTTTAAAATGATTTGAATTATAAATCTTTTAATAAAAGTATTATTAAAACAAAAAAATAA 789
Old-1     TGTGAAATCTATAAAAATTAAATAACTATTTGAAAGAAAACTTTTAAAATGATTTGAATTATAAATCTTTTAATAAAAGTATTATTAAAACAAAAAAATAA 799
Pog-0     TGTGAAATCTATAAAAATTAAATAACTATTTGAAAGAAAACTTTTAA----------------------------------------------------- 715
Ws-0      TGTGAAATCTATAAAAATTAAATAACTATTTGAAAGAAAACTTTTAAAATGATTTGAATTATAAATCTTTTAATAAAAGTATTATTAAWACARAAAATAA 792
Da-1-12   TGTGAAATCTATAAAAATTAAATAACTATTTGAAAGAAAACTTTTAAAATGATTTGAATTATAAATCTTTTAATAAAAGTATTATTAGAACAAAAAAATAA 800
Mz-0      TGTGAAATCTATAAAAATTAAATAACTATTTGAAAGAAAACTTTTAAAATGATTTGAATTATAAATCTTTTAATAAAAGTATTATTAAAACAAAAAAATAA 800
Wa-1      TGTGAAATCTATAAAAATTAAATAACTATTTGAAAGAAAACTTTTAAAATGATTTGAATTATAAATCTTTTAATAAAAGTATTATTAAAACAAAAAAATAA 800
La-0      TGTGAAATCTATAAAAATTAAATAACTATTTGAAAGAAAACTTTTAAAATGATTTGAATTATAAATCTTTTAATAAAAGTATTATTAAAACAAAAAAATAA 800

                 810       820       830       840       850       860       870       880       890       900
          ----|----|----|----|----|----|----|----|----|----|----|----|----|----|----|----|----|----|----|----|
Col-0     AAAATAATGTTTAATTTTACTGAACGAGATCAAATGGTATGTCAGGTGTAAAATATTATAATATATGAAATTTATAACAATTAAAGAAAAATTTGAAAGA 899
C24       AAAATAATGTTTAATTTTACTGAACGAGATCAAATGGTATGTCAGGTGTAAAATATTATAATATATGAAATTTATAACAATTAAAGAAAAATTTGAAAGA 898
Ca-0      AAAATAATGTTTAATTTTACTGAACGAGATCAAATGGTATGTCAGGTATAGAATATTATAATATATGAAATTTATAACAATTAAAGAAAAATTTGAAAGA 900
Gie-0     AAAATAATGTTTAATTTTACTGAACGAGATCAAATGGTATGTCAGGTATAGAATATTATAATATATGAAATTTATAACAATTAAAGAAAAATTTGAAAGA 889
Old-1     AAAATAATGTTTAATTTTACTGAACGAGATCAAATGGTATGTCA-------------------------------------------------------- 843
Pog-0     ---------------------------------------------------------------------------------------------------- 715
Ws-0      AAAATAAWGTTTAATTTTWCTGAACGAGATCAAATGGTATGTCAGGTGTAAAATATTATAATATATGAAATTTATAACAATTAAATAAAAATTTGAAAGA 892
Da-1-12   AAAATAATGTTTAATTTTACTGAACGAGATCAAATGGTATGTCAGGTGTAAAATATTATAATATATGAAATTTATAACAATTAAAGAAAAATTTGAAAGA 900
Mz-0      AAAATAATGTTTAATTTTACTGAACGAGATCAAATGGTATGTCAGGTGTAAAATATTATAATATATGAAATTTATAACAATTAAAGAAAAATTTGAAAGA 900
Wa-1      AAAATAATGTTTAATTTTACTGAACGAGATCAAATGGTATGTCAGGTGTAAAATATTATAATATATGAAATTTATAACAATTAAAGAAAAATTTGAAAGA 900
La-0      AAAATAATGTTTAATTTTACTGAACGAGATCAAATGGTATGTCAGGTATAGAATATTATAATATATGAAATTTATAACAATTAAAGAAAAATTTGAAAGA 900

                 910       920       930       940       950       960       970       980       990      1000
          ----|----|----|----|----|----|----|----|----|----|----|----|----|----|----|----|----|----|----|----|
Col-0     AAACTTTTAAATTGATTTGAATTATAAATTTAAAATATTATTAAAAAAA-TAACAAAATAATGTTTAATTTCACTTAACGAGGTTAGATGGCAGAACGGG 998
C24       AAACTTTTAAATTGATTTGAATTATAAATTTAAAATATTATTAAAAAAA-TAACAAAATAATGTTTAATTTCACTTAACGAGGTTAGATGGCAGAACGGG 997
Ca-0      AAACTTTTAAATTGATTTGAATTATAAATTTAAAATATTATTAAAAAAAATAACAAAATAATGTTTAATTTCACTTAACGAGGTTAGATGGCAGAACGGG 1000
Gie-0     AAACTTTTAAATTGATTTAAATTATAAATTTAAAATATTATTAAAAAAA-TAACAAAATAATGTTTAATTTCACTTAACGAGGTTAGATGGCAGAACGGG 988
Old-1     ----------------TTGAATTATAAATTTAAAATATTATTAAAAAAA-TAACAAAATAATGTTTAATTTCACTTAACGAGGTTAGATGGCAGAACGGG 926
Pog-0     ----------------------------------------TTAAAAAAA-TAACAAAATAATGTTTAATTTCACTTAACGAGGTTAGATGGCAGAACGGG 775
Ws-0      AAACTTTTAAATTGATTTGAATTATAAATTTAAAATATTATTAAAAAAA-TAACAAAATAATGTTTAATTTCACTTAACGAGGTTAGATGGCAGAACGGG 991
Da-1-12   AAACTTTTAAATTGATTTGAATTATAAATTTAAAATATTATTAAAAAAAATAACAAAATAATGTTTAATTTCACTTAACGAGGTTAGATGGCAGAACGGG 1000
Mz-0      AAACTTTTAAATTGATTTGAATTATAAATTTAAAATATTATTAAAAAAAATAACAAAATAATGTTTAATTTCACTTAACGAGGTTAGATGGCAGAACGGG 1000
Wa-1      AAACTTTTAAATTGATTTGAATTATAAATTTAAAATATTATTAAAAAAAATAACAAAATAATGTTTAATTTCACTTAACGAGGTTAGATGGCAGAACGGG 1000
La-0      AAACTTTTAAATTGATTTGAATTATAAATTTAAAATATTATTAAAAAAA-TAACAAAATAATGTTTAATTTCACTTAACGAGGTTAGATGGCAGAACGGG 999
```

Supplementary Figure S2
(Continued)

```
               1010      1020      1030      1040      1050      1060      1070      1080      1090      1100
           ----|----|----|----|----|----|----|----|----|----|----|----|----|----|----|----|----|----|----|----|
Col-0      TTTGAGTCGATAGTAATATGAGACGATAAACCACAGGTGAAATCCATTAGATATGTTTAATTTCACTTAACGGGGTTAGATGGTAGGACGGGTTTGGGTC 1098
C24        TTTGAGTCGATAGTAATATGAGACGATAAACCACAGGTGAAATCCATTAGATATGTTTAATTTCACTTAACGGGGTTAGATGGTAGGACGGGTTTGGGTC 1097
Ca-0       TTTGAGTCGATAGTAATATGAGACGATAAACCACAGGTGAAATCCATTAGATATGTTTAATTTCACTTAACGGGGTTAGATGGTAGAACAGGTTTGGGTC 1100
Gie-0      TTTGAGTCGATAGTAATATGAGACGATAAACCACAGGTGAAATCCATTAGATATGTTTAATTTCACTTAACGGGGTTAGATGGTAGAACAGGTTTGGGTC 1088
Old-1      TTTGAGTCGATAGTAATATGAGACGATAAACCACAGGTGAAATCCATTAGATATGTTTAATTTCACTTAACGGGGTTAGATGGTAGAACAGGTTTGGGTC 1026
Pog-0      TTTGAGTCGATAGTAATATGAGACGATAAACCACAGGTGAAATCCATTAGATATGTTTAATTTCACTTAACGGGGTTAGATGGTAGAACAGGTTTGGGTC 875
Ws-0       TTTGAGTCGATAGTAATATGAGACGATAAACCACAGGTGAAATCCATTAGATATGTTTRRTTTCACTTAACGKGGTTAGATGGTAGGACGGGTTTGGGTC 1091
Da-1-12    TTTGAGTCGATAGTAATATGAGACGATAAACCACAGGTGAAATCCATTAGATATGTTTAATTTCACTTAACGGGGTTAGATGGTAGGACGGGTTTGGGTC 1100
Mz-0       TTTGAGTCGATAGTAATATGAGACGATAAACCACAGGTGAAATCCATTAGATATGTTTAATTTCACTTAACGGGGTTAGATGGTAGGACGGGTTTGGGTC 1100
Wa-1       TTTGAGTCGATAGTAATATGAGACGATAAACCACAGGTGAAATCCATTAGATATGTTTAATTTCACTTAACGGGGTTAGATGGTAGGACGGGTTTGGGTC 1100
La-0       TTTGAGTCGATAGTAATATGAGACGATAAACCACAGGTGAAATCCATTAGATATGTTTAATTTCACTTAACGGGGTTAGATGGTAGGACGGGTTTGGGTC 1099

               1110      1120      1130      1140      1150      1160      1170      1180      1190      1200
           ----|----|----|----|----|----|----|----|----|----|----|----|----|----|----|----|----|----|----|----|
Col-0      GATAGTAAAACGAGACAGTATACCACGAGTGATATACATTAGATATTTTTAATTTTACTGAACGGGATTAATGGTAAGACGGATTTGGATCGATAGTAAT 1198
C24        GATAGTAAAACGAGACAGTATACCACGAGTGATATACATTAGATATTTTTAATTTTACTGAACGGGATTAATGGTAAGACGGATTTGGATCGATAGTAAT 1197
Ca-0       GATAGTAAAACGAGACAGTATACCACGAGTGATATACATTAGATATTTTTAATTTTACTGAACGGGATTAATGGTAAGACGGATTTGGATCGATAGTAAT 1200
Gie-0      GATAGTAAAACGAGACAGTATACCACGAGTGATATACATTAGATATTTTTAATTTTACTGAACGGGATTAATGGTAAGACGGATTTGGATCGATAGTAAT 1188
Old-1      GATAGTAAAACGAGACAGTATACCACGAGTGATATACATTAGATATTTTTAATTTTACTGAACGGGATTAATGGTAAAACGGATTTGGATCGATAGTAAT 1126
Pog-0      GATAATAAAACGAGACAGTATACCACGAGTGATATACATTAGATATTTTTAATTTTACTGAACGGGATTAATGGTAAGACGGATTTGGATCGATAGTAAT 975
Ws-0       GATAGTAAAACGAGACAGTATACCACGAGTGATATACATTAGATATTTTTAATTTTACTGAACGGGATTAATGGTAAGACGGATTTGGATCGATAGTAAT 1191
Da-1-12    GATAGTAAAACGAGACAGTATACCACGAGTGATATACATTAGATATTTTTAATTTTACTGAACGGGATTAATGGTAAGACGGATTTGGATCGATAGTAAT 1200
Mz-0       GATAGTAAAACGAGACAGTATACCACGAGTGATATACATTAGATATTTTTAATTTTACTGAACGGGATTAATGGTAAGACGGATTTGGATCGATAGTAAT 1200
Wa-1       GATAGTAAAACGAGACAGTATACCACGAGTGATATACATTAGATATTTTTAATTTTACTGAACGGGATTAATGGTAAGACGGATTTGGATCGATAGTAAT 1200
La-0       GATAGTAAAACGAGACAGTATACCACGAGTGATATACATTAGATATTTTTAATTTTACTGAACGGGATTAATGGTAAGACGGATTTGGATCGATAGTAAT 1199

               1210      1220      1230      1240      1250      1260      1270      1280      1290      1300
           ----|----|----|----|----|----|----|----|----|----|----|----|----|----|----|----|----|----|----|----|
Col-0      ACGAGACGGTATACCACATGTGAAATCCAAGATATAACAATCAAAATACAATTATTTTATCTTAAATAGTAAACAGAACAAACAATATTAACAAAATAAA 1298
C24        ACGAGACGGTATACCACATGTGAAATCCAAGATATAACAATCAAAATACAATTATTTTATCTTAAATAGTAAACAGAACAAACAATATTAACAAAATAAA 1297
Ca-0       ACGAGACGGTATACCACATGTGAAATCCAAGATATAACAATCAAAATACAATTATTTTATCTTAAATAGTAAACAGAACAAACAATATTAACAAAATAAA 1300
Gie-0      ACGAGACGGTATACCACATGTGAAATCCAAGATATAACAATCAAAATACAATTATTTTATCTTAAATAGTAAACAGAACAAACAATATTAACAAAATAAA 1288
Old-1      ACGAGACGGTATACCACATGTGAAATCCAAGATATAACAATCAAAATACAATTATTTTATCTTAAATAGTAAACAGAACAAACAATATTAACAAAATAAA 1226
Pog-0      ACGAGACGGTATACCACATGTGAAATCCAAGATATAACAATCAAAATACAATTATTTTATCTTAAATAGTAAACAGAACAAACAATATTAACAAAATAAA 1075
Ws-0       ACGAGACGGTATACCACATGTGAAATCCAAGATATAACAATCAAAATACAATTATTTTATCTTAAATAGTAAACAGAACAAACAATATTAACAAAATAAA 1291
Da-1-12    ACGAGACGGTATACCACATGTGAAATCCAAGATATAACAATCAAAATACAATTATTTTATCTTAAATAGTAAACAGAACAAACAATATTAACAAAATAAA 1300
Mz-0       ACGAGACAGTATACCACATGTGAAATCCAAGATATAACAATCAAAATACAATTATTTTATCTTAAATAGTAAACAGAACAAACAATATTAACAAAATAAA 1300
Wa-1       ACGAGACGGTATACCACATGTGAAATCCAAGATATAACAATCAAAATACAATTATTTTATCTTAAATAGTAAACAGAACAAACAATATTAACAAAATAAA 1300
La-0       ACGAGACGGTATACCACATGTGAAATCCAAGATATAACAATCAAAATACAATTATTTTATCTTAAATAGTAAACAGAACAAACAATATTAACAAAATAAA 1299

               1310      1320      1330      1340      1350      1360      1370      1380      1390      1400
           ----|----|----|----|----|----|----|----|----|----|----|----|----|----|----|----|----|----|----|----|
Col-0      TATAATTTAAATTTAAATTATTTAGTTATAATCTAGATCTAGATTTTAACTTAATGAATTGTATGTTATTGTTCCCATACTTTTAAACAGTAAAAATCAA 1398
C24        TATAATTTAAATTTAAATTATTTAGTTATAATCTAGATCTAGATTTTAACTTAATGAATTGTATGTTATTGTTCCCATACTTTTAAACAGTAAAAATCAA 1397
Ca-0       TATAATTTAAATTTAAATTATTTAGTTATAATCTAGATCTAGATTTTAACTTAATGAATTGTATGTTATTGTTCCCATACTTTTAAACAGTAAAAATCAA 1400
Gie-0      TATAATTTAAATTTAAATTATTTAGTTATAATCTAGATCTAGATTTTAACTTAATGAATTGTATGTTATTGTTCCCATACTTTTAAACAGTAAAAATCAA 1388
Old-1      TATAATTTAAATTTAAATTATTTAGTTATAATCTAGATCTAGATTTTAACTTAATGAATTGTATGTTATTGTTCCCATACTTTTAAACAGTAAAA----- 1321
Pog-0      TATAATTTAAATTTAAATTATTTATTTATAATCTAGATCTAGATTTTAACTTAATGAATTGTATGTTATTGTTCCCATACTTTTAAACAGTAAAA----- 1170
Ws-0       TATAATTTAAATTTAAATTATTTAGTTATAATCTAGATCTAGATTWAACTTAATGAATTGTRTGTTATTGTTCCCATACTTTTAAACAGTAAAAATCAA 1391
Da-1-12    TATAATTTAAATTTAAATTATTTAGTTATAATCTAGATCTAGATTTTAACTTAATGAATTGTATGTTATTGTTCCCATACTTTTAAACAGTAAAAATCAA 1400
Mz-0       TATAATTTA------------------------------------------------------------------------------------------- 1307
Wa-1       TATAATTTAAATTTAAATTATTTAGTTATAATCTAGATCTAAATTTTAACTTAATGAATTGTATGTTATTGTTCCCATACTTTTAAACAGTAAAAATCAA 1400
La-0       TATAATTTAAATTTAAATTATTTAGTTATAATCTAGATCTAGATTTTAACTTAATGAATTGTATGTTATTGTTCCCATACTTTTAAACAGTAAAAATCAA 1399

               1410      1420      1430      1440      1450      1460      1470      1480      1490      1500
           ----|----|----|----|----|----|----|----|----|----|----|----|----|----|----|----|----|----|----|----|
Col-0      ACATAGTAATTGTATACTAATATATATATATATATAAGAATTGTACATTTACAAAATACAAAACATTAAATTATATTTTATTATTAACAAATTAAATTTT 1498
C24        ACATAGTAATTGTATACTAATATATATATATATATAAGAATTGTACATTTACAAAATACAAAACATTAAATTATATTTTATTATTAACAAATTAAATTTT 1497
Ca-0       ACATAGTAATTGTATACTAATATATATATATATATAAGAATTGTACATTTACAAAATACAAAACATTAAATTATATTTTATTATTAACAAATTAAATTTT 1500
Gie-0      ACATAGTAATTGTATACTAATATATATATATATATAAG-ATTGTACATTTACAAAATACAAAACATTAAATTATATTTTATTATTAACAAATTAAATTTT 1487
Old-1      ------------------------------TATATAAGAATTGTACATTTACAAAATACAAAACATTAAATTATATTTTATTATTAACAAATTAAATTTT 1391
Pog-0      ------------------------------T-----------GTACATTTACAAAATACAAAACATTAAATTATATTTTATTATTAACAAATTAAATTTT 1229
Ws-0       ACATARTAATTGTATACTAATATATATATATATA--AGAATTGTACATTTACAAAATACAAAACATTAAATTATATTTTATTATTAACAAATTAAATTTT 1489
Da-1-12    ACATAGTAATTGTATACTAATATATATATATATATAAGAATTGTACATTTACAAAATACAAAACATTAAATTATATTTTATTATTAACAAATTAAATTTT 1500
Mz-0       ---------------------------------------------CATTTACAAAATACAAAACATTAAATTATATTTTATTATTAACAAATTAAATTTT 1364
Wa-1       ACATAGTAATTGTATACTAATATATATATATATATAAGAATTGTACATTTACAAAATACAAAACATTAAATTATATTTTATTATTAACAAATTAAATTTT 1500
La-0       ACATAGTAATTGTATACTAATATATATATATATATAAGAATTGTACATTTACAAAATACAAAACATTAAATTATATTTTATTATTAACAAATTAAATTTT 1499
```

Supplementary Figure S2
(Continued)

```
                1510      1520      1530      1540      1550      1560      1570      1580      1590      1600
            ----|----|----|----|----|----|----|----|----|----|----|----|----|----|----|----|----|----|----|----|
Col-0       TGTTTTAAAAAAATAGTTCTACGGTTTACCACGGGTTAAAATCTAGTTGATTTGCATAAATTTAAGCAAGCTCGTTTTTTTATGTTACCATTTAGACCCA 1598
C24         TGTTTTAAAAAAATAGTTCTACGGTTTACCACGGGTTAAAATCTAGTTGATTTGCATAAATTTAAGCAAGCTCGTTTTTTTATGTTACCATTTAGACCCA 1597
Ca-0        TGTTTTAAAAAAATAGTTCTACGGTTTACTACGGGTTAAAATCTAGTTAATTTGCATAAATTTAAGCAAGCTCGTTTTTTTATGTTACCATTTAGACCCA 1600
Gie-0       TGTTTTAAAAAAATAGTTCTACGGTTTACTACGGGTTAAAATCTAGTTGATTTGCATAAATTTAAGCAAGCTCGTTTTTTTATGTTACCATTTAGACCCA 1587
Old-1       TGTTTTAAAAAAATAGTTCTACGGTTTACTACGGGTTAAAATTTAGTTGATTTGCATAAATTTAAGCAAGCTCGTTTTTTTATGTTACCATTTAGACCCA 1491
Pog-0       TGTTTTAAAAAAATAGTTCTACGGTTTACTACGAGTTAAAATCTAGTTGATTTGCATAAATTTAAGCAAGCTCGTTTTTTTATGTTACCATTTAGACCCA 1329
Ws-0        TGTTTTAAAAAAATAGTTCTACGGTTTACCACGGGTTAAAATCTAGTTGATTTGCATAAATTTAAGCAAGCTCGTTTTTTTATGTTACCATTTAGACCCA 1589
Da-1-12     TGTTTTAAAAAAATAGTTCTACGGTTTACCACGGGTTAAAATCTAGTTGATTTGCATAAATTTAAGCAAGCTCGTTTTTTTATGTTACCATTTAGACCCA 1600
Mz-0        TGTTTAAAAAAAATAGTTTTACGGTTTACCACGGGTTAAAATCTAGTTGATTTGCATAAATTTAAGCAAGCTCGTTTTTTTATGTTACCATTTAGACCCA 1464
Wa-1        TGTTTTAAAAAAATAGTTCTACGGTTTACCACGGGTTAAAATCTAGTTGATTTGCATAAATTTAAGCAAGCTCGTTTTTTTATGTTACCATTTAGACCCA 1600
La-0        TGTTTTAAAAAAATAGTTCTACGGTTTACTACGGGTTAAAATCTAGTTGATTTGCATAAATTTAAGCAAGCTCGTTTTTTTATGTTACCATTTAGACCCA 1599

                1610      1620      1630      1640      1650      1660      1670      1680      1690      1700
            ----|----|----|----|----|----|----|----|----|----|----|----|----|----|----|----|----|----|----|----|
Col-0       TAATTATTTTTAAATGCGATCAGGTTTAGTAATATAAATTTGATTATCATTTGATTTACTATATACCTTATGGATACACATGCGTTATTCGTTTTGTAAA 1698
C24         TAATTATTTTTAAATGCGATCAGGTTTAGTAATATAAATTTGATTATCATTTGATTTACTATATACCTTATGGATACACATGCGTTATTCGTTTTGTAAA 1697
Ca-0        TAATTATTTTTAAATGCGATCAGGTTTAGTAATATAAATTTGATTATCATTTGATTTACTATATACCTTATGGATACACATGCGTTATTCGTTTTGTAAA 1700
Gie-0       TAATTATTTTTAAATGCGATCAGGTTTAGTAATATAAATTTGATTATCATTTGATTTACTATATACCTTATGGATACACATGCGTTATTCGTTTTGTAAA 1687
Old-1       TAATTATTTTTAAATGCGATCAGGTTTAGTAATATAAATTTGATTATCATTTGATTTACTATATACCTTATGGATACACATGCGTTATTCGTTTTGTAAA 1591
Pog-0       TAATTATTTTTAAATGCGATCAGGTTTAGTAATATAAATTTGATTATCATTTGATTTACTATATACCTTATGGATACACATGCGTTATTCGTTTTGTAAA 1429
Ws-0        TAATTATTTTTAAATGCGATCAGGTTTAGTAATATAAATTTGATTATCATTTGATTTACTATATACCTTATGGATACACATGCGTTATTCGTTTTGTAAA 1689
Da-1-12     TAATTATTTTTAAATGCGATCAGGTTTAGTAATATAAATTTGATTATCATTTGATTTACTATATACCTTATGGATACACATGCGTTATTCGTTTTGTAAA 1700
Mz-0        TAATTATTTTTAAATGCGATCAGGTTTAGTAATATAAATTTGATTATCATTTGATTTACTATATACCTTATGGATACACATGCGTTATTCGTTTTGTAAA 1564
Wa-1        TAATTATTTTTAAATGCGATCAGGTTTAGTAATATAAATTTGATTATCATTTGATTTACTATATACCTTATGGATACACATGCGTTATTCGTTTTGTAAA 1700
La-0        TAATTATTTTTAAATGCGATCAGGTTTAGTAATATAAATTTGATTATCATTTGATTTACTATATACCTTATGGATACACATGCGTTATTCGTTTTGTAAA 1699

                1710      1720      1730      1740      1750      1760      1770      1780      1790      1800
            ----|----|----|----|----|----|----|----|----|----|----|----|----|----|----|----|----|----|----|----|
Col-0       TCTGTTGCACGTTATCACTTAGCACATTTTGTATTGTGTACACTATATTTATGTGTTTGTATATTCAATTATTTTGTAATTCTTTTATACGTAAACACTA 1798
C24         TCTGTTGCACGTTATCACTTAGCACATTTTGTATTGTGTACACTATATTTATGTGTTTGTATATTCAATTATTTTGTAATTCTTTTATACGTAAACACTA 1797
Ca-0        TCTGTTGCACGTTATCACTTAGCACATTTTGTATTGTGTACACTATATTTATATGTTTGTATATTCAATTATTTTGTAATTCTTTTATACGTAAACACTA 1800
Gie-0       TCTGTTGCACGTTATCACTTAGCACATTTTGTATTGTGTACACTATATTTATATGTTTGTATATTCAATTATTTTGTAATTCTTTTATACGTAAACACTA 1787
Old-1       TCTGTTGCACGTTATCACTTAGCACATTTTGTATTGTGTACACTATATTTATATGTTTGTATATTCAATTATTTTGTAATTCTTTTATACGTAAACACTA 1691
Pog-0       TCTGTTGCACGTTATCACTTAGCACATTTTGTATTGTGTACACTATATTTATATGTTTGTATATTCAATTATTTTGTAATTCTTTTATACGTAAACACTA 1529
Ws-0        TCTGTTGCACGTTATCACTTAGCACATTTTGTATTGTGTACACTATATTTATGTGTTTGTATATTCAATTATTTTGTAATTCTTTTATACGTAAACACTA 1789
Da-1-12     TCTGTTGCACGTTATCACTTAGCACATTTTGTATTGTGTACACTATATTTATGTGTTTGTATATTCAATTATTTTGTAATTCTTTTATACGTAAACACTA 1800
Mz-0        TCTGTTGCACGTTATCACTTAGCACATTTTGTATTGTGTACACTATATTTATGTGTTTGTATATTCAATTATTTTGTAATTCTTTTATACGTAAACACTA 1664
Wa-1        TCTGTTGCACGTTATCACTTAGCACATTTTGTATTGTGTACACTATATTTATGTGTTTGTATATTCAATTATTTTGTAATTCTTTTATACGTAAACACTA 1800
La-0        TCTGTTGCACGTTATCACTTAGCACATTTTGTATTGTGTACACTATATTTATATGTTTGTATATTCAATTATTTTGTAATTCTTTTATACGTAAACACTA 1799

                1810      1820      1830      1840      1850      1860      1870      1880      1890      1900
            ----|----|----|----|----|----|----|----|----|----|----|----|----|----|----|----|----|----|----|----|
Col-0       CACAACGTTTGGGTTATGTATACCTCTATATAAAGACACAACTTGCAACATGCACAATCCACTAAGTTCTATCTTAAAAAGGTCAACTTTAATTCTCACA 1898
C24         CACAACGTTTGGGTTATGTATACCTCTATATAAAGACACAACTTGCAACATGCACAATCCACTAAGTTCTATCTTAAAAAGGTCAACTTTAATTCTCACA 1897
Ca-0        CACAACGTTTGGGTTATGTATACCTCTATATAAAGACACAACTTGCAACATGCACAATCCACTAAGTTCTATCTTAAAAAGGTCAACTTTAATTCTCACA 1900
Gie-0       CACAACGTTTGGGTTATGTATACCTCTATATAAAGACACAACTTGCAACATGCACAATCCACTAAGTTCTATCTTAAAAAGGTCAACTTTAATTCTCACA 1887
Old-1       CACAACGTTTGGGTTATGTATACCTCTATATAAAGACACAACTTGCAACATGCACAATCCACTAAGTTCTATCTTAAAAAGGTCAACTTTAATTCTCACA 1791
Pog-0       CACAACGTTTGGGTTATGTATACCTCTATATAAAGACACAACTTGCAACATGCACAATCCACTAAGTTCTATCTTAAAAAGGTCAACTTTAATTCTCACA 1629
Ws-0        CACAACGTTTGGGTTATGTATACCTCTATATAAAGACACAACTTGCAACATGCACAATCCACTAAGTTCTATCTTAAAAAGGTCAACTTTAATTCTCACA 1889
Da-1-12     CACAACGTTTGGGTTATGTATACCTCTATATAAAGACACAACTTGCAACATGCACAATCCACTAAGTTCTATCTTAAAAAGGTCAACTTTAATTCTCACA 1900
Mz-0        CACAACGTTTGGGTTATGTATACCTCTATATAAAGACACAACTTGCAACATGCACAATCCACTAAGTTCTATCTTAAAAAGGTCAACTTTAATTCTCACA 1764
Wa-1        CACAACGTTTGGGTTATGTATACCTCTATATAAAGACACAACTTGCAACATGCACAATCCACTAAGTTCTATCTTAAAAAGGTCAACTTTAATTCTCACA 1900
La-0        CACAACGTTTGGGTTATGTATACCTCTATATAAAGACACAACTTGCAACATGCACAATCCACTAAGTTCTATCTTAAAAAGGTCAACTTTAATTCTCACA 1899

                1910      1920      1930      1940      1950      1960      1970      1980      1990      2000
            ----|----|----|----|----|----|----|----|----|----|----|----|----|----|----|----|----|----|----|----|
Col-0       AGAAATCNNNATGAGATGTGTTGTTTTGTTTATGGTTTCTTGTCTTCTCATAGTTCTCCTTATAAACCATTTTGAAGGTATTTTACTATTTTCTTTTAAC 1998
C24         AGAAATCNNNATGAGATGTGTTGTTTTGTTTATGGTTTCTTGTCTTCTCATAGTTCTCCTTATAAACCATTTTGAAGGTATTTTACTATTTTCTTTTAAC 1997
Ca-0        AGAAATCNNNATGAGA----TTGTTTTGTTTATGGTTTCTTGTCTTCTCATAGTTCTCCTTATAAACCATTTTGAAGGTATTTTACTATTTTCTTTTAAC 1996
Gie-0       AGAAATCNNNATGAGATGTGTTGTTTTGTTTATGGTTTCTTGTCTTCTCATAGTTCTCCTTATAAACCATTTTGAAGGTATTTTACTATTTTCTTTTAAC 1987
Old-1       AGAAATCNNNATGAGATGTGTTGTTTTGTTTATGGTTTCTTGTCTTCTCATAGTTCTCCTTATAAACCATTTTGAAGGTATTTTACTATTTTCTTTTAAC 1891
Pog-0       AGAAATCNNNATGAGA-GTGT-GTTTTGTTTATGGTTTCTTGTCTTCTCATAGTTCTCCTTATAAACCATTTTGAAGGTATTTTACTATTTTCTTTTAAC 1727
Ws-0        AGAAATCNNNATGAGATGTGTTGTTTTGTTTATGGTTTCTTGTCTTCTCATAGTTCTCCTTATAAACCATTTTGAAGGTATTTTACTATTTTCTTTTAAC 1989
Da-1-12     AGAAATCNNNATGAGATGTGTTGTTTTGTTTATGGTTTCTTGTCTTCTCATAGTTCTCCTTATAAACCATTTTGAAGGTATTTTACTATTTTCTTTTAAC 2000
Mz-0        AGAAATCNNNATGAGATGTGTTGTTTTGTTTATGGTTTCTTGTCTTCTCATAGTTCTCCTTATAAACCATTTTGAAGGTATTTTACTATTTTCTTTTAAC 1864
Wa-1        AGAAATCNNNATGAGATGTGTTGTTTTGTTTATGGTTTCTTGTCTTCTCATAGTTCTCCTTATAAACCATTTTGAAGGTATTTTACTATTTTCTTTTAAC 2000
La-0        AGAAATCNNNATGAGATGTGTTGTTTTGTTTATGGTTTCTTGTCTTCTCATAGTTCTCCTTATAAACCATTTTGAAGGTATTTTACTATTTTCTTTTAAC 1999
```

Supplementary Figure S2
(Continued)

```
                2010      2020      2030      2040      2050      2060      2070      2080      2090      2100
            ----|----|----|----|----|----|----|----|----|----|----|----|----|----|----|----|----|----|----|----|
Col-0       TACAGTGATTGTTATTGGTTCAAGAAATTGGTGTTTCTACAATAACATGCAATATTCGAGTTTGAATTCATGTGGTCACAGAACAATTACAGTATACAAA 2098
C24         TACAGTGATTGTTATTGGTTCAAGAAATTGGTGTTTCTACAATAACATGCAATATTCGAGTTTGAATTCATGTGGTCACAGAACAATTACAGTATACAAA 2097
Ca-0        TACAGTGATTGTTATTGGTTCAAGAAATTGGTGTTTCTACAATAACATGCAATATTCGAGTTTGAATTCATGTGGTCACAGAACAATTACAGTATACAAA 2096
Gie-0       TACAGTGATTGTTATTGGTTCAAGAAATTGGTGTTTCTACAATAACATGCAATATTCGAGTTTGAATTCATGTGGTCACAGAACAATTACAGTATACAAA 2087
Old-1       TACAGTGATTGTTATTGGTTCAAGAAATTGGTGTTTCTACAATAACATGCAATATTCGAGTTTGAATTCATGTGGTCACAGAACAATTACAGTATACAAA 1991
Pog-0       TACAGTGATTGTTATTGGTTCAAGAAATTGGTGTTTCTACAATAACATGCAATATTCGAGTTTGAATTCATGTGGTCACAGAACAATTACAGTATACAAA 1827
Ws-0        TACAGTGATTGTTATTGGTTCAAGAAATTGGTGTTTCTACAATAACATGCAATATTCGAGTTTGAATTCATGTGGTCACAGAACAATTACAGTATACAAA 2089
Da-1-12     TACAGTGATTGTTATTGGTTCAAGAAATTGGTGTTTCTACAATAACATGCAATATTCGAGTTTGAATTCATGTGGTCACAGAACAATTACAGTATACAAA 2100
Mz-0        TACAGTGATTGTTATTGGTTCAAGAAATTGGTGTTTCTACAATAACATGCAATATTCGAGTTTGAATTCATGTGGTCACAGAACAATTACAGTATACAAA 1964
Wa-1        TACAGTGATTGTTATTGGTTCAAGAAATTGGTGTTTCTACAATAACATGCAATATTCGAGTTTGAATTCATGTGGTCACAGAACAATTACAGTATACAAA 2100
La-0        TACAGTGATTGTTATTGGTTCAAGAAATTGGTGTTTCTACAATAACATGCAATATTCGAGTTTGAATTCATGTGGTCACAGAACAATTACAGTATACAAA 2099

                2110      2120      2130      2140      2150      2160      2170      2180      2190      2200
            ----|----|----|----|----|----|----|----|----|----|----|----|----|----|----|----|----|----|----|----|
Col-0       GTACTAGTATGCATGGTCTGTAAAAATAGTATTATGATCAAAGTAATTTCTCAATGATAATTGATGAGACATAAACATTATATTTTTCAATTAGTCATTA 2198
C24         GTACTAGTATGCATGGTCTGTAAAAATAGTATTATGATCAAAGTAATTTCTCAATGATAATTGATGAGACATAAACATTATATTTTTCAATTAGTCATTA 2197
Ca-0        GTACTAGTATGCATGGTCTGTAAAAATAGTATTATGATCAAAGTAATTTCTCAATGATAATTGATGAGACATAAACATTATATTTTTCAATTAGTCATTA 2196
Gie-0       GTACTAGTATGCATGGTCTGTAAAAATAGTATTATGATCAAAGTAATTTCTCAATGATAATTGATGAGACATAAACATTATATTTTTCAATTAGTCATTA 2187
Old-1       GTACTAGTATGCATGGTCTGTAAAAATAGTATTATGATCAAAGTAATTTCTCAATGATAATTGATGAGACATAAACATTATATTTTTCAATTAGTCATTA 2091
Pog-0       GTACTAGTATGCATGGTCTGTAAAAATAGTATTATGATCAAAGTAATTTCTCAATGATAATTGATGAGACATAAACATTATATTTTTCAATTAGTCATTA 1927
Ws-0        GTACTAGTATGCATGGTCTGTAAAAATAGTATTATGATCAAAGTAATTTCTCAATGATAATTGATGAGACATAAACATTATATTTTTCAATTAGTCATTA 2189
Da-1-12     GTACTAGTATGCATGGTCTGTAAAAATAGTATTATGATCAAAGTAATTTCTCAATGATAATTGATGAGACATAAACATTATATTTTTCAATTAGTCATTA 2200
Mz-0        GTACTAGTATGCATGGTCTGTAAAAATAGTATTATGATCAAAGTAATTTCTCAATGATAATTGATGAGACATAAACATTATATTTTTCAATTAGTCATTA 2064
Wa-1        GTACTAGTATGCATGGTCTGTAAAAATAGTATTATGATCAAAGTAATTTCTCAATGATAATTGATGAGACATAAACATTATATTTTTCAATTAGTCATTA 2200
La-0        GTACTAGTATGCATGGTCTGTAAAAATAGTATTATGATCAAAGTAATTTCTCAATGATAATTGATGAGACATAAACATTATATTTTTCAATTAGTCATTA 2199

                2210      2220      2230      2240      2250      2260      2270      2280      2290      2300
            ----|----|----|----|----|----|----|----|----|----|----|----|----|----|----|----|----|----|----|----|
Col-0       AAATAAAAATTTCTAGCAAATGTTTGATTTTTTATTTGTTTATAAGATAATTTCTGGACCTGGTAAAGAGGCATAATTTTAATTTCAAAAGTGATTAATT 2298
C24         AAATAAAAATTTCTAGCAAATGTTTGATTTTTTATTTGTTTATAAGATAATTTCTGGACCTGGTAAAGAGGCATAATTTTAATTTCAAAAGTGATTAATT 2297
Ca-0        AAATAAAAATTTCTAGCAAATGTTTGATTTTTTATTTGTTTATAAGATAATTTCTGGACCTGGTAAAGAGGCATAATTTTAATTTCAAAAGTGATTAATT 2296
Gie-0       AAATAAAAATTTCTAGCAAATTTTTGATTTTTTATTTGTTTATAAGATAATTTCTGGACCTGGTAAAGAGGCATAATTTTAATTTCAAAAGTGATTAATT 2287
Old-1       AAATAAAAATTTCTAGCAAATGTTTGATTTTTTATTTGTTTATAAGATAATTTCTGGACCTGGTAAAGAGGCATAATTTTAATTTCAAAAGTGATTAATT 2191
Pog-0       AAATAAAAATTTCTAGCAAATGTTTGATTTTTTATTTGTTTATAAGATAATTTCTGGACCTGGTAAAGAGGCATAATTTTAATTTCAAAAGTGATTAATT 2027
Ws-0        AAATAAAAATTTCTAGCAAATGTTTGATTTTTTATTTGTTTATAAGATAATTTCTGGACCTGGTAAAGAGGCATAATTTTAATTTCAAAAGTGATTAATT 2289
Da-1-12     AAATAAAAATTTCTAGCAAATGTTTGATTTTTTATTTGTTTATAAGATAATTTCTGGACCTGGTAAAGAGGCATAATTTTAATTTCAAAAGTGATTAATT 2300
Mz-0        AAATAAAAATTTCTAGCAAATGTTTGATTTTTTATTTGTTTATAAGATAATTTCTGGACCTGGTAAAGAGGCATAATTTTAATTTCAAAAGTGATTAATT 2164
Wa-1        AAATAAAAATTTCTAGCAAATGTTTGATTTTTTATTTGTTTATAAGATAATTTCTGGACCTGGTAAAGAGGCATAATTTTAATTTCAAAAGTGATTAATT 2300
La-0        AAATAAAAATTTCTAGCAAATGTTTGATTTTTTATTTGTTTATAAGATAATTTCTGGACCTGGTAAAGAGGCATAATTTTAATTTCAAAAGTGATTAATT 2299

                2310      2320      2330      2340      2350      2360      2370      2380      2390      2400
            ----|----|----|----|----|----|----|----|----|----|----|----|----|----|----|----|----|----|----|----|
Col-0       GATCGAATTAATATTACACAACTAAGATATTTAGAGATATCTACTATATATCTACTTAAGACGAGAATTATCATGATTAATATTAATACCTATCTTTAGC 2398
C24         GATCGAATTAATATTACACAACTAAGATATTTAGAGATATCTACTATATATCTACTTAAGACGAGAATTATCATGATTAATATTAATACCTATCTTTAGC 2397
Ca-0        GATCGAATTAATATTACACAACTAAGATATTTAGAGATATCTACTATATATCTACTTAAGACGAGAATTATCATGATTAATATTAATACCTATCTTTAGC 2396
Gie-0       GATCGAATTAATATTACACAACTAAGATATTTAGAGATATCTACTATATATCTACTTAAGACGAGAATTATCATGATTAATATTAATACCTATCTTTAGC 2387
Old-1       GATCGAATTAATATTACACAACTAAGATATTTAGAGATATCTACTATATATCTACTTAAGACGAGAATTATCATGATTAATATTAATACCTATCTTTAGC 2291
Pog-0       GATCGAATTAATATTACACAACTAAGATATTTAGAGATATCTACTATATATCTACTTAAGACGAGAATTATCATGATTAATATTAATACCTATCTTTAGC 2127
Ws-0        GATCGAATTAATATTACACAACTAAGATATTTAGAGATATCTACTATATATCTACTTAAGACGAGAATTATCATGATTAATATTAATACCTATCTTTAGC 2389
Da-1-12     GATCGAATTAATATTACACAACTAAGATATTTAGAGATATCTACTATATATCTACTTAAGACGAGAATTATCATGATTAATATTAATACCTATCTTTAGC 2400
Mz-0        GATCGAATTAATATTACACAACTAAGATATTTAGAGATATCTACTATATATCTACTTAAGACGAGAATTATCATGATTAATATTAATACCTATCTTTAGC 2264
Wa-1        GATCGAATTAATATTACACAACTAAGATATTTAGAGATATCTACTATATATCTACTTAAGACGAGAATTATCATGATTAATATTAATACCTATCTTTAGC 2400
La-0        GATCGAATTAATATTACACAACTAAGATATTTAGAGATATCTACTATATATCTACTTAAGACGAGAATTATCATGATTAATATTAATACCTATCTTTAGC 2399

                2410      2420      2430      2440      2450      2460      2470      2480      2490      2500
            ----|----|----|----|----|----|----|----|----|----|----|----|----|----|----|----|----|----|----|----|
Col-0       TATATCATAAGAATATTATAATATTCTAAATTAATAAAAATTATTTTATTCCCTTTTACAGAGTTAATTAAGAACAATATACGTCAACCATTAGTAAATA 2498
C24         TATATCATAAGAATATTATAATATTCTAAATTAATAAAAATTATTTTATTCCCTTTTACAGAGTTAATTAAGAACAATATACGTCAACCATTAGTAAATA 2497
Ca-0        TATATCATAAGAATATTATAATATTCTAAATTAATAAAAATTATTTTATTCCCTTTTACAGAGTTAATTAAGAACAATATACGTCAACCATTAGTAAATA 2496
Gie-0       TATATCATAAGAATATTATAATATTCTAAATTAATAAAAATTATTTTATTCCCTTTTACAGAGTTAATTAAGAACAATATACGTCAACCATTAGTAAATA 2487
Old-1       TATATCATAAGAATATTATAATATTCTAAATTAATAAAAATTATTTTATTCCCTTTTACAGAGTTAATTAAGAACAATATACGTCAACCATTAGTAAATA 2391
Pog-0       TATATCATAAGAATATTATAATATTCTAAATTAATAAAAATTATTTTATTCCCTTTTACAGAGTTAATTAAGAACAATATACGTCAACCATTAGTAAATA 2227
Ws-0        TATATCATAAGAATATTATAATATTCTAAATTAATAAAAATTATTTTATTCCCTTTTACAGAGTTAATTAAGAACAATATACGTCAACCATTAGTAAATA 2489
Da-1-12     TATATCATAAGAATATTATAATATTCTAAATTAATAAAAATTATTTTATTCCCTTTTACAGAGTTAATTAAGAACAATATACGTCAACCATTAGTAAATA 2500
Mz-0        TATATCATAAGAATATTATAATATTCTAAATTAATAAAAATTATTTTATTCCCTTTTACAGAGTTAATTAAGAACAATATACGTCAACCATTAGTAAATA 2364
Wa-1        TATATCATAAGAATATTATAATATTCTAAATTAATAAAAATTATTTTATTCCCTTTTACAGAGTTAATTAAGAACAATATACGTCAACCATTAGTAAATA 2500
La-0        TATATCATAAGAATATTATAATATTCTAAATTAATAAAAATTATTTTATTCCCTTTTACAGAGTTAATTAAGAACAATATACGTCAACCATTAGTAAATA 2499
```

Supplementary Figure S2
(Continued)

```
                    2510      2520      2530      2540      2550      2560      2570      2580      2590      2600
            ----|----|----|----|----|----|----|----|----|----|----|----|----|----|----|----|----|----|----|----|
Col-0       CTAAATAGTGAACAATATAATATATATAAGTTGACCATCATAAATAGCTTCCTTTTATCATGTTCTTAAATGTGGAAGGTGTGGTTACTCTCTGTATATGTG 2598
C24         CTAAATAGTGAACAATATAATATATATAAGTTGACCATCATAAATAGCTTCCTTTTATCATGTTCTTAAATGTGGAAGGTGTGGTTACTCTCTGTATATGTG 2597
Ca-0        CTAAATAGTGAACAATATAATATATATAAGTTGACCATCATAAATAGCTTCCTTTTATCATGTTCTTAAATGTGGAAGGTGTGGTTACTCTCTGTATATGTG 2596
Gie-0       CTAAATAGTGAACAATATAATATATATAAGTTGACCATCATAAATAGCTTCCTTTTATCATGTTCTTAAATGTGGAAGGTGTGGTTACTCTCTGTATATGTG 2587
Old-1       CTAAATAGTGAACAATATAATATATATAAGTTGACCATCATAAATAGCTTCCTTTTATCATGTTCTTAAATGTGGAAGGTGTGGTTACTCTCTGTATATGTG 2491
Pog-0       CTAAATAGTGAACAATATAATATATATAAGTTGACCATCATAAATAGCTTCCTTTTATCATGTTCTTAAATGTGGAAGGTGTGGTTACTCTCTGTATATGTG 2327
Ws-0        CTAAATAGTGAACAATATAATATATATAAGTTGACCATCATAAATAGCTTCCTTTTATCATGTTCTTAAATGTGGAAGGTGTGGTTACTCTCTGTATATGTG 2589
Da-1-12     CTAAATAGTGAACAATATAATATATATAAGTTGACCATCATAAATAGCTTCCTTTTATCATGTTCTTAAATGTGGAAGGTGTGGTTACTCTCTGTATATGTG 2600
Mz-0        CTAAATAGTGAACAATATAATATATATAAGTTGACCATCATAAATAGCTTCCTTTTATCATGTTCTTAAATGTGGAAGGTGTGGTTACTCTCTGTATATGTG 2464
Wa-1        CTAAATAGTGAACAATATAATATATATAAGTTGACCATCATAAATAGCTTCCTTTTATCATGTTCTTAAATGTGGAAGGTGTGGTTACTCTCTGTATATGTG 2600
La-0        CTAAATAGTGAACAATATAATATATATAAGTTGACCATCATAAATAGCTTCCTTTTATCATGTTCTTAAATGTGGAAGGTGTGGTTACTCTCTGTATATGTG 2599

                    2610      2620      2630      2640      2650      2660      2670      2680      2690      2700
            ----|----|----|----|----|----|----|----|----|----|----|----|----|----|----|----|----|----|----|----|
Col-0       TATATACCCTCCTTCAAAGTTATCTTCGAAATAATATATATGTGTATAAAACTCTCTAACTATTACTAATCGCAAAATGCAGTATTGCGACAAGAAAAAA 2698
C24         TATATACCCTCCTTCAAAGTTATCTTCGAAATAATATATATGTGTATAAAACTCTCTAACTATTACTAATCGCAAAATGCAGTATTGCGACAAGAAAAAA 2697
Ca-0        TATATACCCTCCTTCAAAGTTATCTTCGAAATAATATAT--GTGTATAAAACTCTCTAACTATTACTAATCGCAAAATGCAGTATTGCGACAAGAAAAAA 2694
Gie-0       TATATACCCTCCTTCAAAGTTATCTTCGAAATAATATATATGTGTATAAAACTCTCTAACTATTACTAATCGCAAAATGCAGTATTGCGACAAGAAAAAA 2687
Old-1       TATATACCCTCCTTCAAAGTTATCTTCGAAATAATATATATGTGTATAAAACTCTCTAACTATTACTAATCGCAAAATGCAGTATTGCGACAAGAAAAAA 2591
Pog-0       TATATACCCTCCTTCAAAGTTATCTTCGAAATAATATATATGTGTATAAAACTCTCTAACTATTACTAATCGCAAAATGCAGTATTGCGACAAGAAAAAA 2427
Ws-0        TATATACCCTCCTTCAAAGTTATCTTCGAAATAATATATATGTGTATAAAACTCTCTAACTATTACTAATCGCAAAATGCAGTATTGCGACAAGAAAAAA 2689
Da-1-12     TATATACCCTCCTTCAAAGTTATCTTCGAAATAATATATATGTGTATAAAACTCTCTAACTATTACTAATCGCAAAATGCAGTATTGCGACAAGAAAAAA 2700
Mz-0        TATATACCCTCCTTCAAAGTTATCTTCGAAATAATATATATGTGTATAAAACTCTCTAACTATTACTAATCGCAAAATGCAGTATTGCGACAAGAAAAAA 2564
Wa-1        TATATACCCTCCTTCAAAGTTATCTTCGAAATAATATATATGTGTATAAAACTCTCTAACTATTACTAATCGCAAAATGCAGTATTGCGACAAGAAAAAA 2700
La-0        TATATACCCTCCTTCAAAGTTATCTTCGAAATAATATATATGTGTATAAAACTCTCTAACTATTACTAATCGCAAAATGCAGTATTGCGACAAGAAAAAA 2699

                    2710      2720      2730      2740      2750      2760      2770      2780      2790      2800
            ----|----|----|----|----|----|----|----|----|----|----|----|----|----|----|----|----|----|----|----|
Col-0       AATAGTTTATATTCTAAAAATTTACTGCATTATCAAGTATTTATCTAAGTATTCTAAAGTAATAACAGAAGTGGAAGCTCAGAAGTGGAACAAGTGCTTT 2798
C24         AATAGTTTATATTCTAAAAATTTACTGCATTATCAAGTATTTATCTAAGTATTCTAAAGTAATAACAGAAGTGGAAGCTCAGAAGTGGAACAAGTGCTTT 2797
Ca-0        AATAGTTTATATTCTAAAAATTTACTGCATTATCAAGTATTTATCTAAGTATTCTAAAGTAATAACAGAAGTGGAAGCTCAGAAGTGGAACAAGTGCTTT 2794
Gie-0       AATAGTTTATATTCTAAAAATTTACTGCATTATCAAGTATTTATCTAAGTATTCTAAAGTAATAACAGAAGTGGAAGCTCAGAAGTGGAACAAGTGCTTT 2787
Old-1       AATAGTTTATATTCTAAAAATTTACTGCATTATCAAGTATTTATCTAAGTATTCTAAAGTAATAACAGAAGTGGAAGCTCAGAAGTGGAACAAGTGCTTT 2691
Pog-0       AATAGTTTATATTCTAAAAATTTACTGCATTATCAAGTATTTATCTAAGTATTCTAAAGTAATAACAGAAGTGGAAGCTCAGAAGTGGAACAAGTGCTTT 2527
Ws-0        AATAGTTTATATTCTAAAAATTTACTGCATTATCAAGTATTTATCTAAGTATTCTAAAGTAATAACAGAAGTGGAAGCTCAGAAGTGGAACAAGTGCTTT 2789
Da-1-12     AATAGTTTATATTCTAAAAATTTACTGCATTATCAAGTATTTATCTAAGTATTCTAAAGTAATAACAGAAGTGGAAGCTCAGAAGTGGAACAAGTGCTTT 2800
Mz-0        AATAGTTTATATTCTAAAAATTTACTGCATTATCAAGTATTTATCTAAGTATTCTAAAGTAATAACAGAAGTGGAAGCTCAGAAGTGGAACAAGTGCTTT 2664
Wa-1        AATAGTTTATATTCTAAAAATTTACTGCATTATCAAGTATTTATCTAAGTATTCTAAAGTAATAACAGAAGTGGAAGCTCAGAAGTGGAACAAGTGCTTT 2800
La-0        AATAGTTTATATTCTAAAAATTTACTGCATTATCAAGTATTTATCTAAGTATTCTAAAGTAATAACAGAAGTGGAAGCTCAGAAGTGGAACAAGTGCTTT 2799

                    2810      2820      2830      2840      2850      2860      2870      2880      2890      2900
            ----|----|----|----|----|----|----|----|----|----|----|----|----|----|----|----|----|----|----|----|
Col-0       CTTAGGGACATTTTCCCTGGGAAATGTGAACATGACGCAAACGCAAAACTACGATGCAAAGAAGACGATGCAAAGAAGACATTAGCGTAAAATACAGCTT 2898
C24         CTTAGGGACATTTTCCCTGGGAAATGTGAACATGACGCAAACGCAAAACTACGATGCAAAGAAGACGATGCAAAGAAGACATTAGCGTAAAATACAGCTT 2897
Ca-0        CTTAGGGACATTTTCCCTGGGAAATGTGAACATGACGCAAACGCAAAACTACGATGCAAAGAAGACGATGCAAAGAAGACATTAGCGTAAAATACAGCTT 2894
Gie-0       CTTAGGGACATTTTCCCTGGGAAATGTGAACATGACGCAAACGCAAAACTACGATGCAAAGAAGACGATGCAAAGAAGACATTAGCGTAAAATACAGCTT 2887
Old-1       CTTAGGGACATTTTCCCTGGGAAATGTGAACATGACGCAAACGCAAAACTACGATGCAAAGAAGACGATGCAAAGAAGACATTAGCGTAAAATACAGCTT 2791
Pog-0       CTTAGGGACATTTTCCCTGGGAAATGTGAACATGACGCAAACGCAAAACTACGATGCAAAGAAGACGAT---AAGAAGACATTAGCGTAAAATACAGCTT 2624
Ws-0        CTTAGGGACATTTTCCCTGGGAAATGTGAACATGACGCAAACGCAAAACTACGATGCAAAGAAGACGATGCAAAGAAGACATTAGCGTAAAATACAGCTT 2889
Da-1-12     CTTAGGGACATTTTCCCTGGGAAATGTGAACATGACGCAAACGCAAAACTACGATGCAAAGAAGACGATGCAAAGAAGACATTAGCGTAAAATACAGCTT 2900
Mz-0        CTTAGGGACATTTACCCTGGGAAATGTGAACATGACGCAAACGCAAAACTACGATGCAAAGAAGACGATGCAAAGAAGACATTAGCGTAAAATACAGCTT 2764
Wa-1        CTTAGGGACATTTACCCTGGGAAATGTGAACATGACGCAAACGCAAAACTACGATGCAAAGAAGACGATGCAAAGAAGACATTAGCGTAAAATACAGCTT 2900
La-0        CTTAGGGACATTTTCCCTGGGAAATGTGAACATGACGCAAACGCAAAACTACGATGCAAAGAAGACGATGCAAAGAAGACATTAGCGTAAAATACAGCTT 2899

                    2910      2920      2930      2940      2950      2960      2970      2980      2990      3000
            ----|----|----|----|----|----|----|----|----|----|----|----|----|----|----|----|----|----|----|----|
Col-0       TAAAATGAATATTTTGTGATAAATTTGTTTTTCTTCAATTAAATTTGTGCTTTAATTATCACTGTTCATAGGTATATAGATTTTGGTTTTGGAGTTGTTA 2998
C24         TAAAATGAATATTTTGTGATAAATTTGTTTTTCTTCAATTAAATTTGTGCTTTAATTATCACTGTTCATAGGTATATAGATTTTGGTTTTGGAGTTGTTA 2997
Ca-0        TAAAATGAATATTTTGTGATAAATTTGTTTTTCTTCAATTAAATTTGTGCTTTAATTATCACTGTTCATAGGTATATAGATTTTGGTTTTGGAGTTGTTA 2994
Gie-0       TAAAATGAATATTTTGTGATAAATTTGTTTTTCTTCAATTAAATTTGTGCTTTAATTATCACTGTTCATAGGTATATAGATTTTGGTTTTGGAGTTGTTA 2987
Old-1       TAAAATGAATATTTTGTGATAAATTTGTTTTTCTTCAATTAAATTTGTGCTTTAATTATCACTGTTCATAGGTATATAGATTTTGGTTTTGGAGTTGTTA 2891
Pog-0       TAAAATGAATATTTTGTGATAAATTTGTTTTTCTTCAATTAAATTTGTGCTTTAATTATCACTGTTCATAGGTATATAGATTTTGGTTTTGGAGTTGTTA 2724
Ws-0        TAAAATGAATATTTTGTGATAAATTTGTTTTTCTTCAATTAAATTTGTGCTTTAATTATCACTGTTCATAGGTATATAGATTTTGGTTTTGGAGTTGTTA 2989
Da-1-12     TAAAATGAATATTTTGTGATAAATTTGTTTTTCTTCAATTAAATTTGTGCTTTAATTATCACTGTTCATAGGTATATAGATTTTGGTTTTGGAGTTGTTA 3000
Mz-0        TAAAATGAATATTTTGTGATAAATTTGTTTTTCTTCAATTAAATTTGTGCTTTAATTATCACTGTTCATAGGTATATAGATTTTGGTTTTGGAGTTGTTA 2864
Wa-1        TAAAATGAATATTTTGTGATAAATTTGTTTTTCTTCAATTAAATTTGTGCTTTAATTATCACTGTTCATAGGTATATAGATTTTGGTTTTGGAGTTGTTA 3000
La-0        TAAAATGAATATTTTGTGATAAATTTGTTTTTCTTCAATTAAATTTGTGCTTTAATTATCACTGTTCATAGGTATATAGATTTTGGTTTTGGAGTTGTTA 2999
```

Supplementary Figure S2
(Continued)

```
              3010      3020      3030      3040      3050      3060      3070      3080      3090
          ----|----|----|----|----|----|----|----|----|----|----|----|----|----|----|----|----|----|----|----|
Col-0     AACCAAGCATTTTTTACAATAGCAAATTCCACCTTTATCAAAAGTTTGACAATTGCATTCAAAAGGGCGAGAGGGTCTGAAATTCTTAGCAAT 3091
C24       AACCAAGCATTTTTTACAATAGCAAATTCCACCTTTATCAAAAGTTTGACAATTGCATTCAAAAGGGCGAGAGGGTCTGAAATTCTTAGCAAT 3090
Ca-0      AACCAAGCATTTTTTACAATAGCAAATTCGACCTTTATCAAAAGTTTGACAATTGCATTCAAAAGGGCGAGAGGGTCTGAAATTCTTAGCAAT 3087
Gie-0     AACCAAGCATTTTTTACAATAGCAAATTCGACCTTTATCAAAAGTTTGACAATTGCATTCAAAAGGGCGAGAGGGTCTGAAATTCTTAGCAAT 3080
Old-1     AACCAAGCATTTTTTACAATAGCAAATTCGACCTTTATCAAAAGTTTGACAATTGCATTCAAAAGGGCGAGAGGGTCTGAAATTCTTAGCAAT 2984
Pog-0     AACCAAGCATTTTTTACAATAGCAAATTCGACCTTTATCAAAAGTTTGACAATCGCATTCAAAAGGGCGAGAGGGTCTGAAATTCTTAGGAAT 2817
Ws-0      AACCAAGCATTTTTTACAATAGCAAATTCCACCTATATCAAAAGTTTGACAATTGCATTCAAAAGGGCGAGAGGGTCTGAAATTCTTAGCAAT 3082
Da-1-12   AACCAAGCATTTTTTACAATAGCAAATTCCACCTTTATCAAAAGTTTGACAATTGCATTCAAAAGGGCGAGAGGGTCTGAAATTCTTAGCAAT 3093
Mz-0      AACCAAGCATTTTTTACAATAGCAAATTCCACCTTTATCAAAAGTTTGACAATTGCATTCAAAAGGGCGAGAGGGTCTGAAATTCTTAGCAAT 2957
Wa-1      AACCAAGCATTTTTTACAATAGCAAATTCCACCTTTATCAAAAGTTTGACAATTGCATTCAAAAGGGCGAGAGGGTCTGAAATTCTTAGCAAT 3093
La-0      AACCAAGCATTTTTTACAATAGCAAATTCGACCTTTATCAAAAGTTTGACAATCGCATTCAAAAGGGCGAGAGGGTCTGAAATTCTTAGCAAT 3092
```

Alignment of nucleotide sequences of *SCR-A* among *A. thaliana* accessions.

Supplementary Figure S2
(Continued)
