# Supplementary material for: Double-Locking Mechanism of Self-Compatibility in Arabidopsis thaliana: The Synergistic Effect of Transcriptional Depression and Disruption of Coding Region in the Male Specificity Gene
Source: Front Plant Sci. 2020 Sep 11;11:576140. doi: 10.3389/fpls.2020.576140 (PMC7517786; doi:10.3389/fpls.2020.576140)
Supplement: Supplementary Table 1 — List of Primers [file DataSheet_1.pdf]

Supplementary Table S1| List of primers

	primer name	sequence (5'-3')
full length of *AhSCR*	AhSCRpro-F	AAGCTTGTTATTAATCTCCAAATTTGAGTCAATC
	AhSCR3’UTR-R	GAGCTCGTGAAAAACATCGTGTAATCGTATTAG
promoter of *AhSCR*	AhSCRpro-F	AAGCTTGTTATTAATCTCCAAATTTGAGTCAATC
	AhSCRpro-R	GGATCCATGTATGTTACAAGTTGTGTCTTTATATAGAGG
promoter of *AtSCR*	AtSCRpro-F	AAGCTTGTAAATCTATAATATATGGGGTCAAATTG
	AtSCRpro-R	GGATCCGTGCATGTTGCAAGTTGTGT
former part of *AhSCR* codin region	AhSCR5’UTR-F	GGATCCAATACAGTAAGCTCTATCTTAAAAAGGTC
	Ah&AtSCRinner-R	CACATATACAGAGAGTAACCACACCTTCC
latter part of *AhSCR* codin region	Ah&AtSCRinner-F	GGAAGGTGTGGTTACTCTCTGTATATGTG
	AhSCR3’UTR-R	GAGCTCGTGAAAAACATCGTGTAATCGTATTAG
former part of *AtSCR* codin region	AtSCR5’UTR-F	GGATCCAATCCACTAAGTTCTATCTTAAAAAGGTC
	Ah&AtSCRinner-R	CACATATACAGAGAGTAACCACACCTTCC
latter part of *AtSCR* codin region	Ah&AtSCRinner-F	GGAAGGTGTGGTTACTCTCTGTATATGTG
	AtSCRinv-R	GAGCTCATTGCTAAGAATTTCAGACCCTCTC
5'-RACE of *SCR-A*	SCR5’race-R	CAAACCATAAACAAAACAACACATCTCAT
3'-RACE of *SCR-A*	SCR3’race-F	GGTGGAATTTGCTATTGTAAAAAATGCTTGGTTTAA
qRT-PCR of *SCR-A*	SCR5’-F	ATGAGATGTGTTGTTTTGTTTATGGTTTC
	SCR5’-R	GTCTTCTTTGCATCGTAGTTTTGCG
qRT-PCR of *SRK-A*	SRK-F	GAAAGAATGTGAAGAGAAGTGCAAG
	SRK-R	GAACAAGCATTAAGCTGATTCCA
18S rRNA	At18SrRNA-F	GGGCATTCGTATTTCATAGTCAGAG
	At18SrRNA-R	CGGTTCTTGATTAATGAAAACATCCT
